# Evolution of Amino Acid Propensities under Stability-Mediated Epistasis

**DOI:** 10.1093/molbev/msac030

**Published:** 2022-02-04

**Authors:** Noor Youssef, Edward Susko, Andrew J Roger, Joseph P Bielawski

**Affiliations:** 1 Department of Systems Biology, Harvard Medical School, Boston, MA, USA; 2 Department of Mathematics and Statistics, Dalhousie University, Halifax, NS, Canada; 3 Department of Biochemistry and Molecular Biology, Dalhousie University, Halifax, NS, Canada; 4 Department of Biology, Dalhousie University, Halifax, NS, Canada

**Keywords:** evolutionary Stokes shift, entrenchment, contingency, stability, protein evolution, amino acid preferences

## Abstract

Site-specific amino acid preferences are influenced by the genetic background of the protein. The preferences for resident amino acids are expected to, on average, increase over time because of replacements at other sites—a nonadaptive phenomenon referred to as the “evolutionary Stokes shift.” Alternatively, decreases in resident amino acid propensity have recently been viewed as evidence of adaptations to external environmental changes. Using population genetics theory and thermodynamic stability constraints, we show that nonadaptive evolution can lead to both positive and negative shifts in propensities following the fixation of an amino acid, emphasizing that the detection of negative shifts is not conclusive evidence of adaptation. By examining propensity shifts from when an amino acid is first accepted at a site until it is subsequently replaced, we find that ≈50% of sites show a decrease in the propensity for the newly resident amino acid while the remaining sites show an increase. Furthermore, the distributions of the magnitudes of positive and negative shifts were comparable. Preferences were often conserved via a significant negative autocorrelation in propensity changes—increases in propensities often followed by decreases, and vice versa. Lastly, we explore the underlying mechanisms that lead propensities to fluctuate. We observe that stabilizing replacements increase the mutational tolerance at a site and in doing so decrease the propensity for the resident amino acid. In contrast, destabilizing substitutions result in more rugged fitness landscapes that tend to favor the resident amino acid. In summary, our results characterize propensity trajectories under nonadaptive stability-constrained evolution against which evidence of adaptations should be calibrated.

## Introduction

Interactions between amino acids within a protein are a fundamental form of epistasis, resulting in amino acid preferences at individual sites that are a function of the complete protein sequence. These interdependencies occur because of functional, structural, or stability constraints on proteins and have significant impact on evolutionary trajectories (Ortlund et al. 2007; Pollock et al. 2012; Gong et al. 2013). It has become evident that accounting for epistasis between sites is critical for explaining various evolutionary dynamics and properties observed in natural sequences (Kimura 1985; Goldstein and Pollock 2017; de la Paz et al. 2020). Here, we focus on epistasis arising due to stability constraints by modeling protein evolution based on first principles of thermodynamics. This modeling framework reproduces realistic properties of proteins with regards to stability values (Goldstein 2011), evolutionary rates (Youssef et al. 2020), temporal and spatial patterns of rate heterogeneity (Goldstein and Pollock 2016), and levels and trends in convergence rates (Goldstein et al. 2015). Using this framework, we explore long-term shifts in amino acid preferences due to nonadaptive stability constraints, where the overall fitness landscape on which the protein evolves remains constant.

Under nonadaptive evolution, the fixed global fitness landscape implies no change in environment and that the function of the protein remains conserved (Wright 1932). Natural selection maintains the protein near a peak on its landscape with equilibrium dynamics shaped by mutation, drift, and selection. At equilibrium, most mutations are deleterious and only a small proportion is beneficial. The higher fixation probability of the fewer but more advantageous mutations is balanced by a lower fixation probability of the more frequent yet disadvantageous mutations. As a result, the proportion of deleterious and beneficial substitutions (i.e., fixed mutations) are equal (Cherry 1998; Goldstein 2013). This scenario contrasts with the dynamics under adaptive evolution where novel protein function or environment lead to shifts in the fitness landscape, rendering the current state suboptimal. Subsequent fixations that increase fitness transiently inflate substitution rates, a key characteristic of adaptive episodes (dos Reis 2015; Jones et al. 2017).

Since its origin, the strictly neutral model of protein evolution is often treated as the null scenario that must be rejected prior to postulating adaptive evolution (Kimura 1968, 1991; Duret 2008). Equilibrium dynamics under stability-constrained models are consistent with nearly-neutral theory (Goldstein 2011). Such stability-constrained models were essential for demonstrating that the marginal stability of many proteins is an expected, emergent phenomena at mutation–drift–selection equilibrium, challenging the notion that evolution actively selects for marginal stability (DePristo et al. 2005; Goldstein 2011). Furthermore, nonadaptive epistatic models predict various characteristics evident in natural proteins—such as marginal stability (Goldstein 2011), as well as differences in mutational tolerance and substitution rates across sites (Youssef et al. 2020)—highlighting that adaptive evolution need not be invoked to explain their presence.

Using a nonadaptive stability-constrained model, Pollock et al. (2012) observed that the preference for a newly substituted amino acid tends to increase over time due to substitutions at other protein sites. They referred to this phenomenon as the “evolutionary Stokes shift” which is underpinned by ensemble entropic effects occurring at the level of the whole sequence (Goldstein and Pollock 2017). Using a different stability model, Shah et al. (2015) performed extensive in silico evolution and observed that substitutions are often contingent on prior replacements at other positions that increased their probability of fixation, and that substitutions were entrenched by subsequent replacements that decreased their rate of reversion. As such, entrenchment and evolutionary Stokes shift have been used interchangeably in the literature (Echave and Wilke 2017; Flynn et al. 2017; Teufel and Wilke 2017; Haddox et al. 2018; Starr et al. 2018; see supplementary table S1, Supplementary Material online for a list of papers where an explicit definition of evolutionary Stokes shift was provided along with direct quotations).

In contrast with these theoretical predictions, experimental evidence suggest that amino acid preferences are often conserved over long evolutionary time scales (Ashenberg et al. 2013; Doud et al. 2015; Risso et al. 2015; Haddox et al. 2018; Starr et al. 2018). More recently, decreases in resident amino acid preferences have been observed (Popova et al. 2019; Stolyarova et al. 2020). The negative shifts in preferences were interpreted as evidence of adaptations to external environmental changes. Specifically, Popova et al. (2019) stated that epistatic interactions between sites “cannot lead to a systematic reduction in fitness of the incumbent alleles, while this is the expected result of fitness changes with origin that is external to the genome.” They referred to this phenomenon as “senescence.”

Faced with seemingly conflicting observations, it is unclear if there are general patterns in how amino acid preferences shift during evolution. Do resident amino acid preferences increase, decrease, or remain conserved? And to what extent are these dynamics shaped by nonadaptive processes? To address these questions, we characterize the trajectories of propensities following an amino acid fixation at a site. Using extensive simulations under a stability-constrained model, we apply two quantitative metrics to evaluate trajectories in propensity (calculated as the expected equilibrium frequency at a given site) over windows of amino acid residency. We observe that all three trajectories emerge from nonadaptive dynamics at mutation–drift–selection equilibrium. Importantly, resident amino acid preferences can decrease merely due to epistatic constraints and in the absence of any adaptive change.

Lastly, we characterize the mechanisms that cause propensities to fluctuate. Following a stabilizing substitution, most sites are more mutationally tolerant. The higher mutational tolerance implies that substitutions will have little or no detriment to fitness. Therefore, the propensities for the resident amino acids decreases. In contrast, destabilizing substitutions result in more restrictive site-specific fitness landscapes, limiting potential substitutions, and increasing the propensity for the resident amino acid. Generally, epistasis tends to conserve the preference for the resident amino acid at a site via a significant negative autocorrelation in propensity changes: increases in propensities tend to be followed by decreases (and vice versa). Importantly, these phenomena emerge from a nonadaptive model of sequence evolution with constraints on protein stability and assuming no external environmental or functional changes.

## Results

### Modeling Approach

Using a thermodynamic model, we equate fitness to the probability of an amino acid sequence, *s*, folding correctly into a native structure at thermodynamic equilibrium, $P_{\text{fold}}(s)$. We define $s\;=\;[a^{1},\;...,\;a^{L}]$ where *a^h^* represents the amino acid present at site *h* and *L* is the length of the protein. Fitness can then be calculated as

(1) $$f(s)=P_{\text{fold}}(s)=\frac{\;\text{exp}[-\beta \Delta G(s)]}{\;\text{exp}[-\beta \Delta G(s)]+1,}$$

where ΔG(s) denotes the thermostability of the sequence, β=1/kT (*kT* = 0.6), *k* is the Boltzmann constant, and *T* is the absolute temperature. We assume no changes in structure or function of the protein so that the global fitness landscape (the mapping between amino acid sequence and fitness) remains constant. Nonetheless, this modeling framework accounts for epistasis by assigning site-specific fitness landscapes dependent on the background sequence (i.e., the amino acids present at all other sites in the protein). The fitness landscape at a site *h* can be fully defined by a vector of length 20, $f^{h}(s)=\{f_{1}^{h}(s),\ldots ,f_{20}^{h}(s)\}$, where $f_{a}^{h}(s)$ is the fitness of the protein given that amino acid *a* occupies site *h* in the context of the background sequence s (where a=1…20 represents the amino acids in arbitrary order). This framework assumes that selection acts only on the final protein product. To account for mutational biases that arise at the DNA level and to account for redundancy in the genetic code, we model the evolution of the codon sequence $s^{c}=[c^{1},\ldots ,c^{L}]$, where *c^h^* represents the codon occupying site *h*, allowing for more realistic mutation dynamics (see Materials and Methods for details).

Amino acids that confer higher fitness values (improve stability) will tend to more frequently occupy a site and have higher expected frequencies (i.e., propensities). The formal relationship between fitness and frequency is

(2) $$\pi_{a}^{h}(s)=\frac{\pi_{a}^{\left(0\right)}e^{2N_{e}f_{a}^{h}(s)}}{\sum_{x}\pi_{x}^{\left(0\right)}e^{2N_{e}f_{x}^{h}(s),}}$$

where *N*_e_ is the effective population size and $\pi {}_{a}^{\left(0\right)}$ are the neutral stationary frequencies (dos Reis 2015). We calculate $\pi_{a}^{\left(0\right)}$ as the sum over the neutral stationary frequencies for synonymous codons for each amino acid. The site-specific frequency landscape at site *h*, given a particular background sequence *s*, is defined by as a vector of length 20 $\pi^{h}(s)=\{\pi_{1}^{h}(s),\ldots ,\pi_{20}^{h}(s)\}$, representing the frequency for each amino acid.

We equate propensity to the expected frequency, as described by equation (2), which represents a theoretical quantity at equilibrium and assumes that the rest of the sequence is held constant. In previous work (Pollock et al. 2012; Goldstein and Pollock 2017), propensities represented thermodynamic preferences of amino acids and effectively did not permit mutational biases or codon redundancy. This can be accommodated in our formulation by assuming a uniform $\pi_{a}^{\left(0\right)}$ distribution ($\pi_{a}^{\left(0\right)}$ = 1/20). However, our simulations are based on natural proteins with unequal nucleotide frequencies and transition/transversion rate biases. We account for these by approximating protein-specific $\pi_{a}^{\left(0\right)}$ values based on the nucleotide frequencies and mutation rates estimated from multiple sequence alignments for each of the proteins (see Materials and Methods for details; supplementary fig. S1, Supplementary Material online). Nevertheless, our results remained consistent under both definitions of propensity (supplementary fig. S2 and table S2, Supplementary Material online). Unless otherwise stated, we use the mutation-biased expected frequencies to measure amino acid propensities because it can be directly estimated from sequence alignments of natural proteins. Importantly, we observed that the fittest amino acid does not necessarily have the highest propensity. This occurs when a suboptimal amino acid has many codon aliases—the high number of synonymous codons and/or mutational bias can increase the residue’s frequency despite its lower fitness.

The results presented below are based on 500 protein-specific simulations based on three natural proteins. The proteins differ in structure (PDB codes: 1qhw, 2ppn, and 1pek), function (a phosphatase, an isomerase, and a proteinase), and length (300, 107, and 279 amino acids). We ran each simulation for 500 substitutions with *N*_e_ = 100. These simulation parameters allow for extensive diversity: On average, sequences diverged at 43% of sites within a simulation. Increasing the number of substitutions (5,000 substitutions) or the effective population size ($N_{e}=10^{6}$) did not alter our results (supplementary table S2 and supplementary text, Supplementary Material online).

### Increases, Decreases, and Conservation of Preferences under Nonadaptive Evolution

**Fig. 1.**
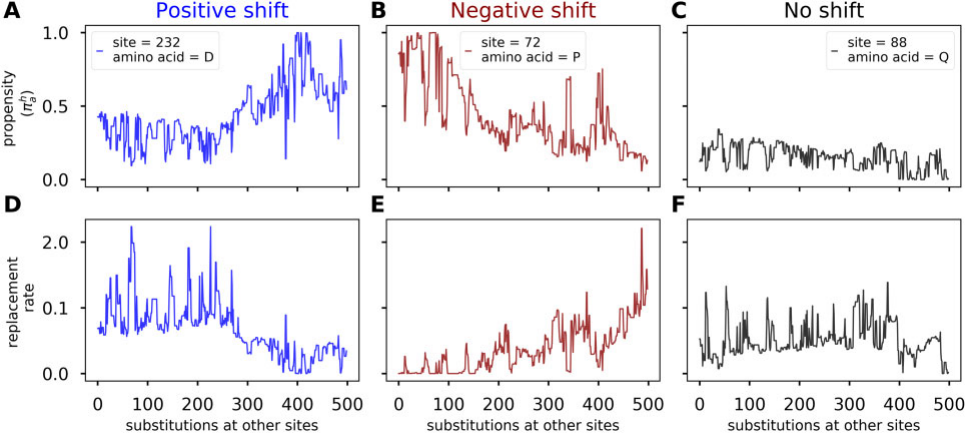
Trajectories of amino acid preferences under nonadaptive evolution. (*A*, *B*, *C*) Trajectories of resident amino acid propensities as substitutions occur at other positions in the protein. (*D*, *E*, *F*) Trajectories of expected replacement rates calculated as the sum of transition rates to neighboring sequences that differ from the current sequence at the site of interest. (*A*, *D*) The propensity for the resident amino acid at site 232, aspartic acid (one letter code *D*), increases over time. (*B*, *E*) The propensity for the resident amino acid at site 72, proline (one letter code P), decreases over time. (*C*, *F*) The propensity for the resident amino acid glutamine (one letter code Q) at site 88 remains conserved. Results are from a simulation of the 1pek protein.

Throughout our simulations, and in natural protein evolution (Ashenberg et al. 2013; Gong et al. 2013; Risso et al. 2015; Youssef et al. 2021), the preference for amino acids fluctuate. In natural proteins, these variations can occur because of nonadaptive processes (such as epistasis) or adaptive processes (such as environmental or functional changes). By contrast, variations in site-specific fitness and propensity landscapes in our simulations are due solely to stability-induced epistasis and are not adaptive. Examples of these propensity dynamics are shown in figure 1. The propensity for aspartic acid (D), the resident amino acid at site 232, increases as substitutions occur at other sites (fig. 1*A*). Alternatively, at site 72 the propensity for the resident amino acid proline (P) decreases (fig. 1*B*), whereas at site 88, the propensity for glutamine (Q), the resident amino acid, remains relatively conserved (fig. 1*C*). All three trajectories emerged at mutation–drift–selection equilibrium and in the absence of adaptive changes.

Shifts in amino acid propensities can be assessed experimentally by comparing the fitness effect of an amino acid mutation in the context of different background sequences, or shifts can be inferred from analyses of multiple sequence alignments (see Youssef et al. [2021] for a review). In particular, shifted propensities will lead to variations in replacement rates (Popova et al. 2019; Gelbart and Stern 2020). Therefore, in addition to the amino acid propensities, we calculated the expected replacement rate confirming the predicted effect (Figure 1D and E). At site 232, the increase in propensity is accompanied by a decrease in the expected replacement rate (fig. 1*A* and *D*). Similarly, the decrease in resident amino acid propensity at site 72, is accompanied by an increase in the expected replacement rate (fig. 1*B* and *E*). Therefore, both increases and decreases in replacement rates can occur under nonadaptive evolution.

### A Balance in Frequency and Magnitude of Positive and Negative Shifts in Amino Acids Preferences Following Substitution

**Fig. 2.**
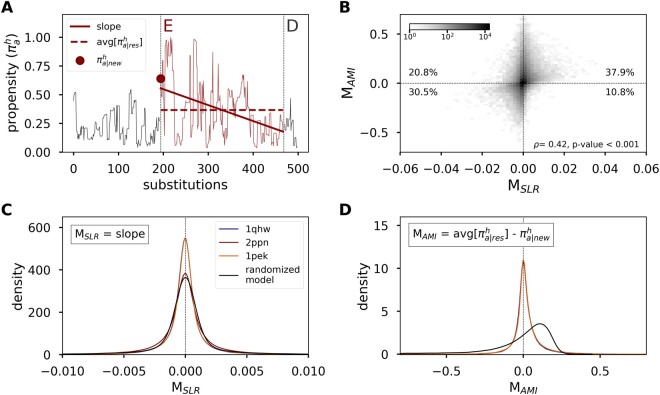
Description of metrics used to estimate propensity shifts. (*A*) Plotted is an example trajectory observed at site 82 of the 1pek protein. The site accepts two substitutions (vertical dotted lines) and the resident amino acid changes from D →E →D. We focus on the dynamics following the acceptance of amino acid E. The first metric, *M*_SLR_, is the Slope of the Linear Regression where *x* is the number of substitutions and *y* is the propensity of the resident amino acid *a* at site *h* ($\pi_{a}^{h}$) calculated over i≤x≤j; *i* is the substitution where amino acid *a* first occupies the site and *j* is the last substitution. The second metric *M*_AMI_ is the Average propensity of an amino acid while it is resident (avg[$\pi_{a|\text{res}}^{h}$]) Minus its Initial propensity ($\pi_{a|\text{new}}^{h}$). Metric values > 0 indicate positive shifts in resident amino acid propensities and values < 0 indicate negative shifts in propensities. (*B*) Hexbin plot showing the relationship between *M*_SLR_ and *M*_AMI_. The shade of each hexbin represents the number of points per hexbin. Reported are the relative percentage of points within each quadrant across all simulations. (*C*, *D*) The distribution of *M*_SLR_ and *M*_AMI_, respectively, estimated from 500 simulations for each of three proteins (1qhw, 2ppn, and 1pek), and the distributions based on a randomized model where propensities changed randomly over time.

The previous results demonstrate that propensity shifts can occur under nonadaptive evolution. However, it remains unclear whether shifts are widespread or rare. To address this, we developed two metrics to quantify trends in propensities over windows where an amino acid is first accepted and subsequently replaced at a site. The metrics are described in detail in the Materials and Methods and illustrated in figure 2*A*. Briefly, metric *M*_SLR_ is the Slope of the Linear Regression where the covariate *x* is time (measured in substitutions) and the response *y* is the propensity of the resident amino acid. Although propensity values are expected to correlate (Pollock et al. 2012), this is not expected to bias the slopes of different trajectories to be positive or negative. *M*_SLR_ is therefore an adequate metric for identifying the average trend in propensity. Additionally, we quantify shifts using a second metric, *M*_AMI_, calculated as the Average propensity of an amino acid while it is resident Minus its Initial propensity. This metric is consistent with the sitewise dynamics that are expected to occur under the evolutionary Stokes shift where “the inherent propensity for [an] amino acid at that position will be, on average, higher than it was when the substitution occurred” (Pollock et al. 2012). Values of *M*_SLR_ and M_AMI_>0 indicate a positive shift in the propensity of the resident amino acid, whereas values < 0 suggest a decrease in the resident amino acid propensity.

Given the time reversibility of the underlying evolutionary model (Goldstein and Pollock 2017), any set of sequences observed forward in time is, in the long run, expected to be observed backward in time and with the same frequency. Thus, a set of sequences where the propensity for the resident amino acid increases is also expected to be observed in the reverse direction with negative propensity trends. Therefore, in theory, positive and negative shifts should be balanced. However, because sequence space is so vast, and movement on it is highly correlated, such arguments need not apply over relatively short evolutionary time scales (or timescales comparable with those observed in natural proteins). In practice, therefore, it is unlikely that one would observe the same set of sequences in forward and reverse order.

Results from *M*_SLR_ reflect the expected balance in positive and negative shifts (fig. 2C and table 1). However, estimates from *M*_AMI_ suggest an excess of positive shifts (≈60%) compared with negative shifts (≈40%; fig. 2*D* and table 1). Why do percentages differ under *M*_SLR_ and *M*_AMI_? And, more importantly, is the excess of positive shifts under *M*_AMI_ due to epistatic adjustments at other sites? A natural null model to investigate these dynamics is a model of neutral evolution (e.g., Halpern and Bruno [1998]). However, under such a model propensities would not vary at all because sites are assumed to evolve independently and hence have fixed site-specific landscapes. Therefore, an alternative model in which both positive and negative shifts are expected to occur with equal frequencies is required to assess the discrepancy in the metrics and whether the estimates may be biased. To this end, we developed two models, an autocorrelated and a randomized model, where the resident and initial propensity were drawn from the same distribution and hence no systematic trend in propensities to either increase or decrease during an amino acid’s residency is expected. Both models sampled initial and resident amino acid propensities from the empirical propensity distribution observed in the stability simulations. In the autocorrelated model, the sampling ensured that the propensities in this simulation had the same first-order autocorrelation as was observed in the stability simulations (*R* = 0.95). In the randomized model, propensities were sampled randomly from the empirical distribution, allowing us to assess the behavior of the system in the absence of any epistatic or temporal adjustments. In both these simulations, *M*_SLR_ estimated equal percentages for positive and negative shifts (table 1). However, *M*_AMI_ estimated a higher percentage of positive shifts compared with negative shifts. The higher estimated occurrence of positive shifts, therefore, does not suggest that positive shifts are truly occurring at higher frequencies but rather highlights a statistical artifact associated with *M*_AMI_.

**Table 1.** Percentage of Negative Shifts, Where the Propensity for the Resident Amino Acid Decreases during Its Residency ($M_{X}<0$ for X = SLR or AMI).

	*M*_SLR_	*M*_AMI_
1qhw	51.8	42.4
2ppn	51.0	41.0
1pek	50.9	40.8
Randomized model	49.0	34.6
Autocorrelated model	46.8	42.0

Note.—Results are based on 500 protein-specific simulations (1qhw, 2ppn, and 1pek), a randomized model where propensities changed randomly over time, and an autocorrelated model where propensities had the same first-order autocorrelation as observed in the simulation.

**Fig. 3.**
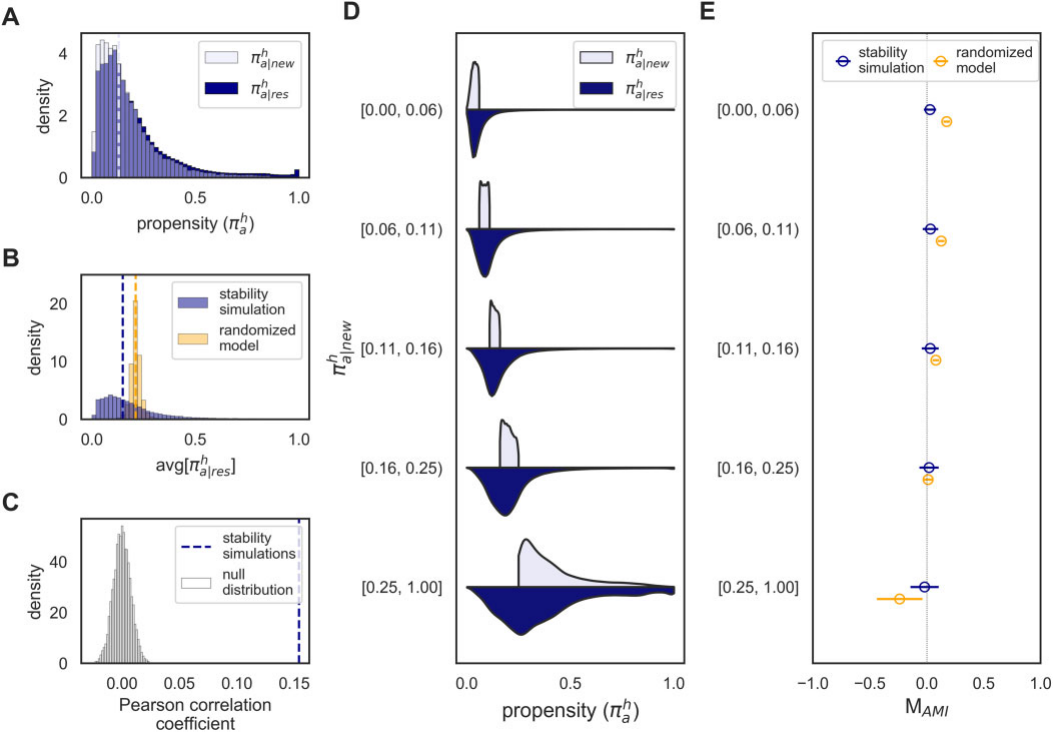
Stability-mediated epistasis conserves amino acid preferences. (*A*) Empirical distribution of initial ($\pi_{a|\text{new}}^{h}$) and resident ($\pi_{a|\text{res}}^{h}$) amino acid propensities observed during simulations of the 1qhw protein. Dotted line represents the median $\pi_{a|\text{new}}^{h}$ value. (*B*) Distribution of the average propensity of an amino acid while it is resident in the stability simulation (blue distribution) and the randomized model where propensities varied randomly over time (yellow distribution). Lines represent the median value from the respective distribution. (*C*) Pearson correlation between propensities of previously resident amino acids ($\pi_{\text{old}}^{h}$) and newly accepted residues ($\pi_{\text{new}}^{h}$) observed in the simulations (blue line) compared with a null distribution (gray distribution). The null distribution was obtained by estimating the correlation between the propensity of the previous amino acid ($\pi_{\text{old}}^{h}$) and the propensity of a randomly sampled residue given the same site and background sequence. This was repeated 10,000 times. (*D*) Violin plots showing the distributions of $\pi_{a|\text{res}}^{h}$ (dark blue) given that $\pi_{a|\text{new}}^{h}$ (light blue) was within a specific range. Ranges were selected such that approximately 20% of substitutions occurred within each range. (*E*) The mean and standard deviation for *M*_AMI_ estimates within each $\pi_{a|\text{new}}^{h}$ range.

We can understand the cause of this kind of artifact by evaluating the distribution of amino acid propensities. Propensities are often less than 0.5 when an amino acid is first substituted, and in most cases, they remain low (fig. 3*A*). The distribution of an average of sampled propensities from such a distribution will not be the same as the distribution of a single (initial) propensity (fig. 3*A* compared with fig. 3*B*). We, therefore, hypothesize that the asymmetry in the propensity distribution is leading to higher estimates of positive shifts under *M*_AMI_. To test this hypothesis, we simulated trajectories by sampling resident amino acid propensities from two symmetric distributions: a uniform distribution *U*(0,1) and a truncated normal distribution *N*(0.5, 0.1). For consistency, we sampled residency times from the empirical distribution of times observed during the stability-constrained simulations. When the propensity distribution was symmetric (normal or uniform), both metrics, *M*_AMI_ and *M*_SLR_, estimated equal proportions of positive and negative shifts (supplementary figs. S3 and S4, Supplementary Material online). This demonstrates that *M*_AMI_ is sensitive to the shape of the propensity distribution and will estimate an excess of increases in resident amino acid propensities if the distribution is asymmetric.

Although the higher occurrence of positive shift under *M*_AMI_ can be explained by the shape of the propensity distribution observed in the simulation, an important question remains: Why are propensities so often less than 0.5 in the stability simulation? Substitutions tend to occur within a “neutral zone” where the original and newly substituted amino acids have similar fitness contributions, and therefore similar propensity values (Goldstein and Pollock 2017). This is evident from the higher correlation between propensities of the original and newly substituted amino acids than the correlation between the original amino acid and other residues (fig. 3*C*). Because all 20 amino acid propensities must sum to one, and the propensities for the original and newly substituted amino acids must be similar, they are likely to be ≤0.5. Although this explains why the distribution of *initial* propensities is right skewed (with most of the density less than 0.5), it is unclear why the propensities after acceptance and while the amino acid is *resident* are similarly distributed (fig. 3*A*). We explore this in detail below.

### Stability-Mediated Epistasis Conserves, Rather Than Alters, Amino Acid Propensities

It is initially surprising that amino acids tend to have similar propensities when they are resident compared with when they are first accepted at a site since it seems to counter the expectation from the evolutionary Stokes shifts where it is expected that “epistatic interactions [will] cause shifts in amino acid preferences, tending to make the newly resident amino acid more favorable” (Pollock and Goldstein 2014). However, Pollock et al. (2012) previously observed that the rate of decay for amino acids preferences at a site is relatively slow. By looking at autocorrelations in propensities as a function of the number of substitutions, they observed that initially high autocorrelation values decay over time consistent with a stretched exponential. This autocorrelation in propensities leads to a distribution of resident amino acid propensities that tends to closely match the distribution of initial propensities (fig. 3*D*).

Although resident and initial propensities were comparable in the stability simulations, average propensities were often higher than initial propensities in the randomized model (compare median lines in fig. 3*A* and *B*). Furthermore, the percentages of positive shifts from the randomized model were higher than those from the stability simulations under *M*_AMI_ (table 1) and the median value was an order of magnitude higher in the random model (5e-2) compared with the stability simulation (7e-3; fig. 2*D*). In other words, resident amino acid propensities were more likely to increase if propensities were changing randomly compared with stability-constrained evolution.

In the stability simulations, an amino acid having a high initial propensity is likely to continue enjoying high propensity throughout its residency, and low initial propensities often remain low (fig. 3*D*). When initial propensities were between 0.0 and 0.06, there were fewer instances of positive shift in the stability simulation than the randomized model, leading to a lower average *M*_AMI_ value (fig. 3*E*). In contrast, when initial propensities were high, between 0.25 and 1.0, there were fewer instances of negative shifts in the stability simulation than the null model, leading to a higher average *M*_AMI_ value (fig. 3*E*). This further supports that propensities are more conserved in the stability simulations than the randomized model.

That resident amino acid propensities were more conserved in the stability simulations is expected given the autocorrelations in amino acid propensities (Pollock et al. 2012). In addition to this, we observed a significant negative first-order autocorrelation in propensity changes (autocorrelation averaged across sites were between −0.21 and −0.24; supplementary table S3, Supplementary Material online). This suggests that increases in propensity tend to be followed by decreases (and vice versa) leading to lower variability in propensities in the stability model compared with the expectation if propensities were varying randomly. Although these results suggest that stability-mediated epistasis frequently conserves amino acid propensities, there will be instances where propensities shift considerably over time at some sites. Importantly, however, nonadaptive dynamics will be balanced in the frequencies and magnitudes of such increases and decreases in propensities, consistent with a nearly-neutral system where, on average, each beneficial substitution is balanced by a corresponding deleterious substitution (Hartl and Taubes 1996; Sella and Hirsh 2005).

### The Dynamics of Positive and Negative Shifts in Propensities Are Comparable under Nonadaptive Evolution

A drawback of the current metrics is that they cannot distinguish between different underlying propensity dynamics. For example, the metrics estimate similar values for the following scenarios: 1) a rapid increase (or decrease) in amino acid propensity followed by a longer period where the propensity remains high (or low), and 2) a more gradual increase (or decrease) in propensity over time. It may be the case that positive shifts occur soon after a substitution, whereas negative shifts are more gradual. To quantify whether propensity changes accelerated or decelerated, we compared the absolute value of each metric calculated over the first half of the amino acid residency (we label this as *M*1_X_) and the estimate over the second half (*M*2_X_), where X is either SLR or AMI. Specifically, we calculated the rate of propensity change as $(|M2_{X}|-|M1_{X}|)/T_{\text{res}}$ where *T*_res_ is the amino acid residency time (measured in number of substitutions). If the dynamics are such that there is an initial rapid change (increase or decrease) in propensity, then the rate of change will be greater than zero. Alternatively, a consistent and gradual shift in propensity will result in a rate of change that is approximately zero. We found no significant differences in the average rates of change between positive and negative shifts (Welch’s *t*-test, all *P* values > 0.05, supplementary table S4, Supplementary Material online). Additionally, we observed that amino acids that are physicochemically similar to the resident amino acid will tend to experience similar shifts in propensities (both negative and positive; supplementary fig. S5, Supplementary Material online).

### Shifts in Propensities Occur at Exposed and Buried Sites

A site’s location in a protein influences its evolutionary dynamics. For globular proteins, surface residues are usually involved with protein function (e.g., binding affinity, enzymatic activity) with a preference for hydrophilic residues, whereas buried sites prefer hydrophobic residues and evolve slower (Shahmoradi et al. 2014; Yeh et al. 2014; Echave et al. 2015; Marcos and Echave 2015). Two measures of a site’s location in the protein are relative solvent accessibility (RSA) and weighted contact number (WCN). Both RSA and WCN correlate significantly with substitution rates in natural (Shahmoradi et al. 2014; Yeh et al. 2014; Marcos and Echave 2015) and simulated proteins (Youssef et al., 2020). Exposed sites have higher substitution rates, higher RSA, and lower WCN than buried sites. In line with these observations, we found a negative correlation between average residency time and RSA and a positive correlation with WCN (supplementary fig. S6, Supplementary Material online).

Popova et al. (2019) recently suggested that buried sites are more likely to undergo positive shifts in propensities of resident amino acids, whereas exposed sites are more prone to decreases in propensities. We assessed sites’ susceptibility to positive and negative shifts by examining the relationship between the metrics and location in the protein. The metrics show no tendency to increase (or decrease) with either RSA (supplementary fig. S7, Supplementary Material online) or WCN (supplementary fig. S8, Supplementary Material online). Furthermore, the average values of *M*_SLR_ at exposed and buried sites were not significantly different (Welch’s *t*-test, *P* values > 0.05 for all proteins; supplementary table S5, Supplementary Material online. Conditional distributions shown in supplementary fig. S9, Supplementary Material online). Although the average *M*_AMI_ values were significantly higher at buried compared with exposed sites (Welch’s *t*-test, *P* values < 0.001 for all proteins; supplementary table S5, Supplementary Material online), the effect sizes were minor (6e-3, 1e-2, and 8e-3 for the 1qhw, 1pek, and 2ppn proteins, respectively). Therefore, positive and negative shifts do not tend to be associated with the locations of sites in a protein. Our conclusions are consistent with experimental results in the HIV envelope protein where sites with shifted propensities were observed across the protein (Haddox et al. 2018).

Next, we assessed whether location in the protein might influence the rate of propensity changes. For example, a deleterious substitution at a surface site might be compensated for by adjustments at a small number of interacting sites, leading to a rapid shift in propensity. Alternatively, a deleterious substitution at a highly connected site might require more adjustments at other positions, and, therefore, the propensity shift may be gradual. However, we found that the average rates of change were not significantly different at buried and exposed sites (supplementary fig. S10 and table S6, Supplementary Material online).

### Stabilizing Substitutions Increase Resident Amino Acid Propensities whereas Destabilizing Substitutions Decrease Them

**Fig. 4.**
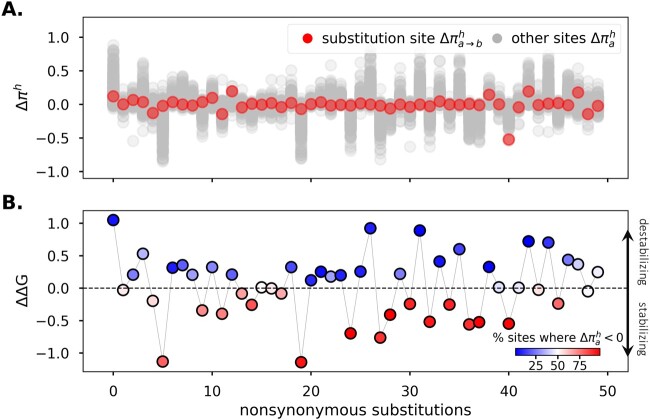
Stabilizing substitutions reduce resident amino acid propensities while destabilizing substitutions often increase propensities. (*A*) Stability-mediated epistasis between sites results in changes in resident amino acid propensities as substitutions accrue. Following an amino acid replacement at one position in the protein, so that the sequence changes from $s_{x}\to s_{x+1}$, the propensity of the resident amino acids at all sites will change. The gray dots are the changes in the propensities of the resident amino acids at each site in the protein following a substitution, $\Delta \pi_{a}^{h}=\pi_{a}^{h}(s_{x+1})-\pi_{a}^{h}(s_{x})$. The red dots are the change in the propensity of the resident amino acid at the substitution site, and therefore a change in the amino acid from a→b ($\Delta \pi_{a\to b}^{h}=\pi_{b}^{h}(s_{x+1})-\pi_{a}^{h}(s_{x})$). (*B*) Stabilizing substitutions (ΔΔG<0) decrease resident amino acid propensities at most sites. In contrast, destabilizing substitutions (ΔΔG>0) result in a lower percentage of sites where $\Delta \pi_{a}^{h}<0$.

We have shown that long-term shifts in amino acid preferences can occur because of nonadaptive stability-mediated epistasis. Next, we turn to the underlying mechanisms that cause changes in propensities after a single substitution. Following a substitution, the fitness and propensity landscapes at most sites in the protein will change because of epistasis. Important questions about how substitutions alter propensities remain unanswered: Do substitutions tend to favorably impact some sites (by increasing their resident amino acid propensities) while simultaneously disadvantaging other sites (by decreasing their resident amino acid propensities)? Or does a substitution impact propensities similarly across sites? We found that the effect of substitution on resident amino acid propensities is unbalanced. Substitutions either favorably (or disfavorably) impact most sites by increasing (or decreasing) their resident amino acid propensity (fig. 4*A*). In particular, stabilizing substitutions (ΔΔG<0) were associated with decreases in propensities of resident amino acids at most sites while destabilizing substitutions (ΔΔG>0) caused propensities to increase (fig. 4*B*). This result is a consequence of the higher stability sequences occupying flatter regions in sequence space such that a higher fraction of mutations is effectively neutral, consistent with experimental findings (Gong et al. 2013).

**Fig. 5.**
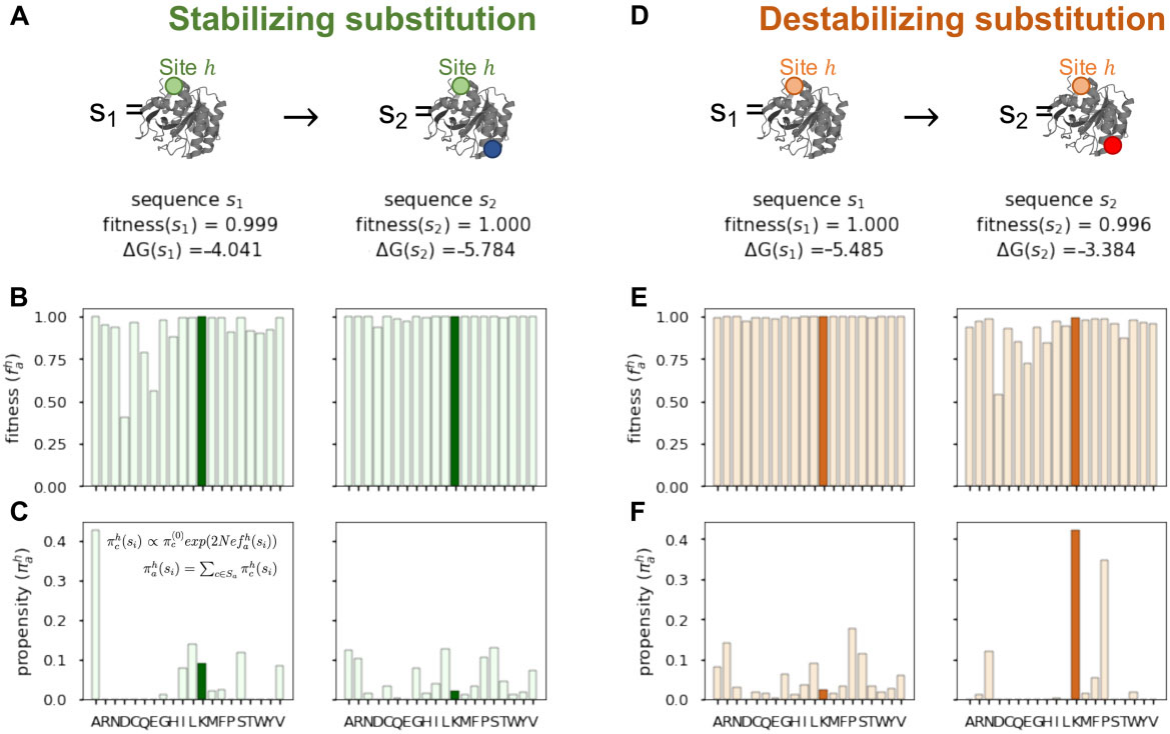
Epistatic dynamics following the fixations of stabilizing and destabilizing substitutions. (*A*) Let *s*_1_ be the initial protein sequence, and *s*_2_ be the sequence following the acceptance of a stabilizing substitution (blue dot). Given the stability-buffered sequence *s*_2_, deleterious mutations which would not have been fixed in *s*_1_ are now more likely to be fixed (e.g., R, N, P). The fitness landscape (*B*) and propensity (*C*) landscapes at a nonsubstituted site 145 becomes more uniform. The fitness and propensity of the resident amino acid is shown in dark green. The propensity for the resident amino acid decreases as the landscapes become flatter. (*D*), (*E*), and (*F*) are the respective plots following the fixation of a destabilizing substitution (red dot). The fitness and propensity landscapes at the nonsubstituted site become less uniform, and the propensity for the resident amino acid increases. These landscapes were observed in simulations of the 1pek protein.

To illustrate the effect, consider the dynamics following a stabilizing substitution from $s_{1}\to s_{2}$ (fig. 5). We focus on site 145 as an example of the site-specific dynamics. The uphill move from *s*_1_ to *s*_2_ (fig. 5*A*) flattened the fitness landscape at site 145 (fig. 5*B*). Given that sequence *s*_2_ has greater stability, a destabilizing mutation has a smaller fitness effect relative to the same mutation in the less stable *s*_1_ sequence. How does the change in the fitness landscape relate to variations in propensities? Because a higher number of amino acids can now occupy the site with little or no detriment to protein fitness, the propensity landscape will similarly become more uniform (fig. 5*C*). Amino acids like R, N, and P that had low propensity in the context of sequence *s*_1_, are more likely given the “stability-buffered” sequence *s*_2_ (fig. 5*C*). Because propensities must sum to one, the increase in the propensity of some amino acids (e.g., R, N, and P) will cause a decrease in the propensity of the resident amino acid (K in this example). The opposite trends are evident following the fixation of a destabilizing mutation (fig. 5*D*). The fitness and propensity landscapes became less uniform (fig. 5*E* and *F*), with fewer amino acids having nonzero propensities, and an increase in resident amino acid propensities.

**Fig. 6.**
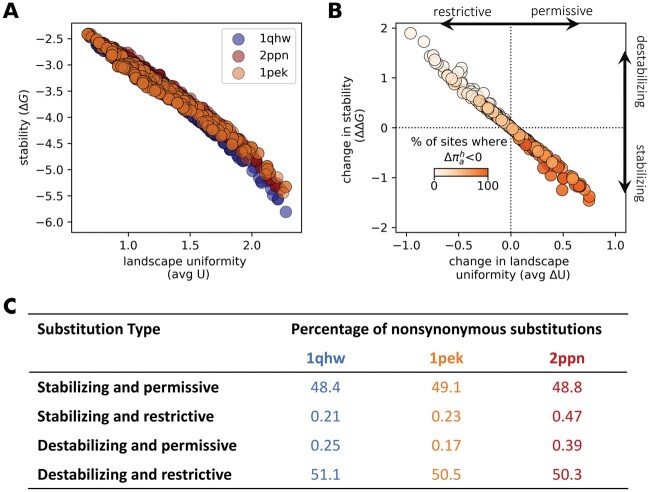
Stabilizing substitutions are permissive and destabilizing substitutions are restrictive. (*A*) The relationship between protein stability (ΔG) and landscape uniformity, measured as the entropy of the propensity landscape averaged over all sites in the protein (avg *U*). (*B*) The relationship between the stability effect of a substitutions (ΔΔG) and the resulting average change in landscape uniformity (avg Δ*U*). Color bar represents the percentage of sites for which the propensity for the resident amino acid decreased ($\Delta \pi_{a}^{h}<0$). Positive avg Δ*U* values imply that, on average, the landscapes became more uniform. Therefore, the substitution is deemed permissive. Negative avg Δ*U* are indicative of restrictive substitutions. Plotted results are based on a single simulation of the 1pek protein. (*C*) The percentages of different types of substitutions for each of three proteins (1qhw, 2ppn, and 1pek). Percentages are calculated from 500 protein-specific simulations.

To quantify the effect across all sites, we measured landscape uniformity using the Shannon entropy $U^{h}(s)$ (see Materials and Methods for detail). Entropy is highest (≈3) when the landscape is uniform (i.e., all amino acids have equal frequencies) and is at a minimum (=0) when only one amino acid has a nonzero propensity. Note that the uniformity of fitness and propensity landscapes are highly correlated (supplementary fig. S11, Supplementary Material online). The fitness landscape describes the fitness of nearby sequences, whereas propensity landscapes consider how frequently nearby sequences are explored. We, therefore, report the entropy of the propensity landscapes, although we expect similar results based on fitness landscapes. As expected, at higher stability values (lower ΔG), the landscapes were more uniform compared with at lower stability values (fig. 6*A*, Spearman correlation coefficients <−0.98 for all proteins, *P* values < 0.001).

Next, we assessed how substitutions alter landscape uniformity. A change from a uniform to a rugged landscape (with a small number of amino acids having nonzero propensities), will result in a negative Δ*U^h^*. In contrast, a positive Δ*U^h^* indicates an increase in landscape uniformity. We considered a substitution as permissive if, on average, it increased landscape uniformity across sites (i.e., a positive average Δ*U*). A restrictive substitution is one where following its acceptance, the landscapes at most sites permit fewer amino acids (i.e., a negative average Δ*U*). The stability effect of a substitution (ΔΔG) is strongly correlated with its influence on landscape uniformity (fig. 6*B*, Spearman correlation coefficient −0.99, *P* value < 0.001). Consistent with the results in figure 5, stabilizing substitutions provide a stability-buffered background so that slightly destabilizing mutations are more likely to be fixed, expanding the space of potential evolutionary paths (fig. 6*C*). In contrast, destabilizing substitutions were restrictive, limiting potential evolutionary trajectories (fig. 6*C*).

## Discussion

We have examined the evolutionary dynamics of proteins under nonadaptive stability constraints. We found that as proteins become more stable, adverse fitness effects of mutations diminish thereby expanding the space of potential evolutionary trajectories. It has been suggested that highly stable proteins may be more adaptable to new functions because they are more likely to accept destabilizing, yet functionally beneficial, mutations than less stable proteins (Schreiber et al. 1994; Wang et al. 2002; DePristo et al. 2005; Nagatani et al. 2007). We suggest that highly stable proteins, all other things being equal, may also be more adaptable because they are more apt to explore neighboring regions of sequence space. It is important to note that selection on other properties of proteins, such as their expression level and the cost of translation error (Drummond et al. 2005), can also influence their evolution. Therefore, the relationship between evolvability and stability of proteins is likely to reflect the complex interplay of multiple factors.

As more (or fewer) mutations become accessible, the propensity for the resident amino acid at a site will change. Stabilizing substitutions expand evolutionary paths and, in doing so, decrease resident amino acid propensities (figs. 4 and 6). By contrast, destabilizing substitutions limit accessible trajectories and favor the resident amino acid. At mutation–selection–drift equilibrium, the proportion of stabilizing and destabilizing substitutions is expected to be equal (Hartl and Taubes 1996; Cherry 1998; Sella and Hirsh 2005; Goldstein 2011), leading to a balance in the proportion of increases and decreases in propensities (supplementary fig. S12, Supplementary Material online). This balance may manifest as 1) an equal number of propensity increases and decreases for any given site; or 2) some sites undergoing systematic increases in propensities, whereas others undergo systematic decreases in propensities. Our results favor the former scenario because fluctuations in propensities were negatively autocorrelated and there was no inherent tendency for sites to experience positive versus negative shifts. Thus, fitness and propensity fluctuations following a substitution at a site due to epistatic background effects, over long evolutionary timescales (as many as 5,000 substitutions), are expected to be constrained. This suggests that dynamics will often be largely a consequence of marginal fitness effects at sites, which is consistent with findings in Youssef et al. (2021) about evolutionary rate variation.

Evolutionary Stokes shift, contingency, entrenchment, and senescence describe phenomena that occur as proteins evolve (Pollock et al. 2012; Shah et al. 2015; Popova et al. 2019). How these phenomena relate to each other is vague and their use in the literature is often inconsistent (supplementary table S1, Supplementary Material online). Here, we aim to clarify these terms and their relationship to each other. Contingency and entrenchment describe changes in the relative fixation probabilities of substitutions, whereas evolutionary Stokes shifts and senescence relate to changes in the propensities of resident amino acids. Because both fixation probabilities and amino acid propensities are a function of the fitness landscape, these concepts are interlinked. Increases in resident amino acid propensities are associated with lower reversion rates over time. As such, evolutionary Stokes shift and entrenchment have often been used interchangeably (Echave and Wilke 2017; Flynn et al. 2017; Teufel and Wilke 2017; Haddox et al. 2018; Starr et al. 2018). Similarly, decreases in the propensity of the resident amino acid entails concomitant increases in both propensities of nonresident amino acids and their probabilities of fixation (i.e., contingency). Because of this, we have previously referred to decreases in resident amino acid propensities as an evolutionary anti-Stokes shift (Youssef et al. 2021). The previously described anti-Stokes shift refers to propensity trajectories where the propensity for the resident amino acid decreases during its residency because of replacements at other positions. This site-level phenomena emerges from thermodynamic stability constraints which are influenced by entropic effects occurring at the sequence level (Goldstein and Pollock 2017). In contrast with these phenomena, that can arise under *nonadaptive* epistatic processes, senescence was used to denote decreases in resident amino acid propensities due to external *adaptive* changes (Popova et al. 2019; Stolyarova et al. 2020). However, as we have clearly demonstrated here, resident amino acid propensities can decrease in the absence of any external environmental change.

Variation in replacement rates over time have been inferred as a proxy for shifting amino acid preferences: increases in resident amino acid propensity lead to decreases in replacement rates, and decreases in propensity lead to higher replacement rates (Popova et al. 2019; Gelbart and Stern 2020; Stolyarova et al. 2020). Analysis of natural protein alignments often reveal a balance in the number of rate accelerating and decelerating sites. For example, across five mitochondrial genes 21/28 sites showed evidence of replacement rate decreases/increases (Stolyarova et al. 2020), and 137/134 sites across nine proteins in HIV and SIV (Gelbart and Stern 2020). Popova et al. (2019) analyzed four influenza A protein alignments and found that the ratios of replacement rate decreases/increases were 2/0, 0/0, 4/12, and 5/8 for the H1, N2, H3, and N2 proteins, respectively. In light of the results presented here, the balance in rate increases and decreases in these data sets is suggestive of nonadaptive processes, contrary to arguments made in the aforementioned papers (Popova et al. 2019; Stolyarova et al. 2020). Importantly, however, the excess of rate increases in the H3 protein could indicate evidence of adaptation to an evolving host immune response (Popova et al. 2019). Future work assessing the dynamics of propensity shifts under adaptive evolution is warranted because it is important to calibrate our evidence of adaptations with nonadaptive signals. Our argument follows a broader trend towards evaluating adaptive dynamics against more realistic null scenarios derived from nearly-neutral models (Hartl and Taubes 1996; Goldstein 2011; Razeto-Barry et al. 2012; Jones et al. 2017, 2020; Tamuri and dos Reis 2021).

An advantage of thermodynamic stability models is that they provide plausible nonadaptive null models for protein evolution (Goldstein 2011; Pollock et al. 2012; Goldstein and Pollock 2017). They have been used to critically assess adaptationist claims about the trade-offs between protein function and stability (Taverna and Goldstein 2002; Goldstein 2011), and protein function and foldability (Govindarajan and Goldstein 1996). “Despite the seduction of adaptive rationalizations,” to quote one of the original authors of this model, “neutral evolutionary dynamics remains the null model that must first be rejected” (Goldstein 2011). Our demonstration that amino acid propensities may decrease over time in the absence of external environmental changes does not preclude that environmental shifts could render resident amino acids less favorable. Rather our simulations demonstrate that decreases in propensities are expected to occur in the absence of external changes, and therefore that their mere occurrence should not, on their own, be taken as conclusive evidence of adaptations.

## Materials and Methods

### Descriptions of Natural Proteins

We simulated the evolution of three proteins with PDB codes 1qhw, 2ppn, and 1pek. The proteins differ in structure, function, length, and contact density. The 1qhw protein is a phosphatase, the 1pek protein is a proteinase, and the 2ppn protein is an isomerase. The 1qhw protein has 300 amino acids, 1pek is made of 297 amino acids, and the 2ppn protein comprises 107 residues. The 1pek protein was the most densely packed with an average number of contacts per site of 8.4 compared with 7.5 for the 1qhw protein and 6.9 for the 2ppn protein.

### Evolutionary Model

The evolutionary process is based on the mutation–selection (MutSel) framework (Halpern and Bruno 1998), assuming a Wright–Fisher population with fixed effective population size (*N*_e_) under a weak mutation, strong selection regime so that only a single variant exists in the population at any time point. Given a current sequence *s*, the probability that sequence *t*, with a single mutated site, will be fixed in a diploid population is

(3) $$P_{\text{fix}}=\frac{1-\text{exp}[-2\{f(t)-f(s)\}]}{1-\text{exp}[-4N_{e}\{f(t)-f(s)\}],}$$

where *f*(*s*) is the fitness of sequence *s*. Selection is assumed to act on the final protein product, and therefore all synonymous codons have the same fitness. We performed simulations with *N*_e_ = 100 and 10^6^. Both yielded similar results (supplementary table S2 and supplementary text, Supplementary Material online). Unless otherwise stated, we report on results from *N*_e_ = 100 simulations in the main text.

We model the substitution process as a continuous-time Markov chain that is specified by the instantaneous rate matrix *Q* with elements

(4) $$q_{\text{st}}\propto 2N_{e}\mu_{\text{st}}P_{\text{fix}}$$

where *q*_st_ is the substitution rate from sequence *s* to *t* which depends on the mutation rate (*μ*_st_) and the fixation probability (*P*_fix_). We model mutations at the DNA level following the HKY85 model (Hasegawa et al. 1985) allowing only single nucleotide changes. The mutation rate depends on the newly substituted nucleotide at the codon site where *t* differs from *s*. Specifically, the mutation rate is given by:

(5) $$\mu_{\text{st}}\propto \{\begin{array}{cc} \pi_{n} & \;\text{if}\;s\;\text{and}\;t\;\text{differ by}\;a\;\text{transversion}\\ \kappa \pi_{n} & \;\text{if}\;s\;\text{and}\;t\;\text{differ by}\;a\;\text{transition} \end{array}$$

where *κ* is the transition–transversion rate ratio and *π_n_* is the stationary frequency of the substituted nucleotide *n*. During the simulations, we used the nucleotide frequencies (*π_n_*) and transition/transversion rate (*κ*) parameters estimated from multiple sequence alignments for the corresponding protein used in Youssef et al. (2020). The mutation parameters ($\kappa ,\pi_{A},\pi_{C},\pi_{G},\pi_{T}$) were set equal to (4.37, 0.21, 0.32, 0.28, 0.20) for the 1qhw protein; (0.90, 0.19, 0.35, 0.56, 0.21) for the 1pek protein; and (2.50, 0.27, 0.24, 0.29, 0.19) for the 2ppn protein.

The state space of a sequence model is made up of 20^*L*^ possible states where *L* is the length of the protein. For all but the smallest proteins, it is impossible to calculate the $20^{L}\times 20^{L}$ substitution matrix P(t)=exp[Qt] for use in simulation. However, given that the process is currently at sequence *s*, it is feasible to calculate the transition rates to all single-nucleotide step neighboring sequences. To simulate the process of sequence evolution, the probability of a transition into another state given that the process is currently at state *s* is calculated as

(6) $$P_{\text{st}}=\frac{q_{\text{st}}}{\sum_{t\neq s}q_{\text{st}.}}$$

At each time step, the substitution to the next state *t* is determined by a random draw from a multinomial distribution with probabilities *P*_st_.

To initiate our simulations, we used the algorithm outlined in supplementary table S7, Supplementary Material online to obtain protein sequences with fitness values ≥ 0.99 given the corresponding protein structure. Then, we evolved the equilibrated sequence for 500 substitutions while keeping track of the site-specific fitness landscapes at all sites. The reported results are based on the post-equilibration phase. We generated 500 protein-specific replicates for each protein. We assume equilibrium is reached if ΔG values were approximately constant (as done in Ashenberg et al. [2013] and Pollock et al. [2012]). Allowing for a longer equilibration yielded similar results (supplementary table S2, Supplementary Material online). Additionally, increasing the number of substitutions per simulation had no effect on the reported results (supplementary table S2 and supplementary text, Supplementary Material online).

### Stability Model

We use the same stability model outlined in Goldstein (2011), Pollock et al. (2012), Goldstein and Pollock (2017), and Youssef et al. (2020). We assume that the fitness, *f*(*s*) for a sequence *s*, is equal to the probability of it being in the native structure at thermodynamic equilibrium, $P_{\text{fold}}(s)$ as defined in equation (1). Thermostability is the difference in free energy between the folded (*E*_F_) and unfolded states (*E*_U_), $\Delta G=E_{F}-E_{U}$. The free energy of a sequence *s* in a given structure *k* is approximated as the sum of pairwise potentials for amino acids in contact.

(7) $$E_{k}=\sum_{x<y}\varepsilon_{\text{MJ}}(a^{x},a^{y})\;{\text{CM}}_{k}^{x,y,}$$

where $\varepsilon_{\text{MJ}}$ are the contact potentials determined by Miyazawa and Jernigan (1985), and CM_*k*_ is the contact matrix specifying interactions between sites in structure *k* such that ${\text{CM}}_{k}^{x,y}=1$ if site *x* and *y* are in contact and 0 otherwise. Residues are considered to be in contact if the C${}_{\beta }$ atoms are within 7 Å of each other. If the amino acid present is glycine, distance is considered with reference to the C${}_{\alpha }$ atom. The free energy associated with the folded state *E*_F_ can be calculated using equation (7), with *k* as the known native structure. Usually, however, there is not a single unfolded configuration and it is intractable to characterize the entire set of possible unfolded structures, making it challenging to estimate *E*_U_ directly. Instead, a subset of structures $\left\{k_{U}\right\}$ is selected to characterize the distribution of thermodynamic properties of the ensemble of unfolded microstates (supplementary table S8, Supplementary Material online). Then, the free energy in the unfolded state is given by the Helmholtz equation:

(8) $$E_{U}=-\beta^{-1}\ln Z_{U}$$

where β=1/kT(=1/0.6), *k* is the Boltzmann constant, *T* is absolute temperature, and *Z*_U_ is the partition function. Assuming that the free energies approximately follow a Gaussian distribution, the set of structures $\left\{k_{U}\right\}$ is used to estimate the mean $\bar{E}$ and standard deviation $\Delta E^{2}$ that define the distribution, ρ(E), of free energies, *E*, over unfolded states:

(9) $$\rho (E)=\frac{1}{\sqrt{2\pi \Delta E^{2}}}\;\;\text{exp}\;[\frac{-{\left(E-\bar{E}\right)}^{2}}{2\Delta E^{2}}].$$

The partition function sums over all unfolded energies which is equivalently a sum of all possible energies, weighted by how frequently they arise:

(10) $$Z_{U}=\sum_{i=1}^{N_{U}}\;\text{exp}\;(-\beta E_{i})$$

(11) $$\;\approx N_{U}\int \rho (E)\;\text{exp}(-\beta E)dE$$

(12) $$\;=N_{U}\;\;\text{exp}\;(\frac{1}{2}\beta^{2}\Delta E^{2}-\beta \bar{E}),$$

where *N*_U_ is the number of unfolded microstates set equal to 3.4^*L*^, allowing for 3.4 configurations per residue. Finally, the stability of a sequence *s* can be rewritten as

(13) $$\Delta G=E_{F}+\beta^{-1}\ln Z_{U}$$

(14) $$\;=E_{F}-\bar{E}+\frac{1}{2}\beta \Delta E^{2}+\beta^{-1}\ln N_{U}$$

### Amino Acid Propensities

Suppose that for a simulation trial we observed $s_{1}\to s_{2}\to \ldots \to s_{500}$ where the *s_x_*’s are the codon sequences realized during the simulations, and *s_x_* and $s_{x+1}$ differ by a single nucleotide substitution (synonymous or nonsynonymous). Given the translated amino acid sequence $s=[a^{1},\ldots ,a^{L}]$, we can calculate the fitness of any amino acid *b* at site *h* holding the rest of the sequence constant, $f_{b}^{h}(s)=f(a^{1},\ldots ,a^{h-1},b^{h},a^{h+1},\ldots ,a^{L})$. The fitness landscape at a site can then be fully defined by a 20-element vector $f^{h}(s)=\{f_{1}^{h}(s),\ldots ,f_{20}^{h}(s)\}$ for each of the 20 amino acids in arbitrary order. We use these fitness values and neutral amino acid frequencies, $\pi_{a}^{\left(0\right)}$, to calculate propensities using equation (2). The $\pi_{a}^{\left(0\right)}$ are calculated as the sum over the neutral stationary frequencies for synonymous codons for each amino acid. The neutral frequency for a codon made up of nucleotide triplet *lmn* is proportional to $\pi_{l}\pi_{m}\pi_{n}$.

### Description of Metrics Used to Quantify Shifts in Propensities

We define two metrics to quantify shifts in propensities. First, let the residence time of an amino acid (*T*_res_) be the time period between *i* and *j*, where *i* is the substitution when amino acid *a* first occupies the site and *j* is the last substitution. The first metric is the Slope of the Linear Regression over *T*_res_ where the covariate *x* is time (measured in substitutions) and the response *y* is the propensity of the resident amino acid *a* at site *h* ($\pi_{a}^{h}$). We refer to this metric as *M*_SLR_. The second metric *M*_AMI_ is the difference in the Average propensity of an amino acid while it is resident (avg[$\pi_{a|\text{res}}^{h}]$) Minus its Initial propensity ($\pi_{a|\text{new}}^{h}$). Metrics values greater than 0 are suggestive of positive shifts in amino acid propensities and values less than 0 are indicative of negative shifts. Figure 2 provides a visual representation of the metrics.

For all results described in this study, we only considered the dynamics when a residue was accepted and subsequently replaced within the timeframe of the simulation, and where the amino acid was resident for at least ten substitutions. However, we repeated the analyses with the inclusion of partial windows (where for example an amino acid is accepted during the simulation but the simulation ends prior to its replacement) which revealed similar results.

### Quantifying the Uniformity of a Landscape

We use the Shannon entropy of a propensity landscape as a measure of its uniformity. We calculate entropy as

(15) $$U^{h}(s)=-\sum_{a}\pi_{a}^{h}(s)\ln\pi_{a}^{h}(s),$$

where $\pi_{a}^{h}(s)$ is the propensity of amino acid *a* at site *h* given background sequence *s*. The entropy is maximized (≈3) when all amino acids are equally likely, and is minimized (= 0) when only a single amino acid is observed. To determine how the landscapes change in response to changes in the background protein sequence, we compared the entropy before and after the substitution

(16) $$\Delta U^{h}=H^{h}(s_{x+1})-H^{h}(s_{x}).$$

We classified a substitution as permissive if the average Δ*U* across all sites was positive, and restrictive if the average Δ*U* was negative.

### The Rate of Amino Acid Replacement

We calculate the rate of leaving the resident amino acid at a site *h* as the sum of the transition rates (using eq. 4) over all sequences that differ from the current sequence by a single nucleotide and have a different amino acid at site *h*.

### Autocorrelated and Randomized Models

We developed two models, an autocorrelated and a randomized model, to examine the behavior of the metrics, *M*_SLR_ and *M*_AMI_, in the absence of any systematic trend for propensities to increase or decrease. First, we sampled 10,000 window sizes (i.e., residency times) from the empirical distribution observed in the stability simulations. In the randomized model, we randomly sampled propensity values from the empirical propensity distribution plotted in figure 3*A* over a given window size. For the autocorrelated model, we sampled from the empirical distribution in such a way as to ensure that the propensities were autocorrelated to a similar extent as in the stability simulations. Importantly, in both models, initial and resident amino acid propensities were sampled from the same distribution and therefore no systematic increase (or decrease) in propensity is expected. Then, we estimated *M*_SLR_ and *M*_AMI_ for each window. The estimated percentages of positive and negative shifts based on each measure are reported in table 1. The algorithm used to generate the autocorrelated data (and a proof that the marginal empirical distribution of propensities matched the empirical distribution) are provided in the supplementary text, Supplementary Material online.

## Acknowledgements

Funding for this research was provided by the Natural Sciences and Engineering Research Council of Canada (NSERC) Discovery Grant program awarded to E.S. (DG04287), A.R (DG06792), and J.P.B (DG04109).

## Supplementary Material

Supplementary data are available at *Molecular Biology and Evolution* online.

## Data Availability

All codes used to simulate, analyze, and plot data have been uploaded and are freely available from https://github.com/nooryoussef/antiStokes_shifts (last accessed February 17, 2022).

## Supplementary Material

msac030_Supplementary_Data
