# Supplementary material for: Evolution of Amino Acid Propensities under Stability-Mediated Epistasis
Source: Mol Biol Evol. 2022 Feb 4;39(3):msac030. doi: 10.1093/molbev/msac030 (PMC8896634; doi:10.1093/molbev/msac030)
Supplement: msac030_Supplementary_Data [file msac030_supplementary_data.zip › Supp_figures_and_tables.pdf]

Supplementary Tables:

Table S1. Definitions of evolutionary Stokes shifts. We systematically reviewed every paper that cited Pollock *et al.,* 2012, a total of 107 papers (citations were extracted from Web of Science, SCOPUS and PubMed from April 2012 until February 15th 2021). Below are papers where a definition was provided along with direct quotations.

Paper	Direct Quotation
Pollock et al 2012	Here, our most notable finding is the existence of what we call an evolutionary Stokes shift, by which we mean that, on substitution of an amino acid at a position in a protein, the protein will tend to adjust through coevolutionary processes to having that amino acid at that position; therefore, the inherent propensity for that amino acid at that position will be, on average, higher than it was when the substitution occurred.
Sandler et al 2012	Pollock et al. used molecular simulation to reveal that following amino acid replacement, proteins tend to equilibrate and adapt to the substitution(s), leading to a changed propensity of accepting further amino acid replacements.
Ashenberg et al 2013	Pollock et al. (2) have used computer simulations to suggest that there are widespread evolutionary shifts in mutational effects, where for instance a mutation is destabilizing in one homolog but stabilizing in another homolog due to interactions with other covarying sites.
Pollock and Goldstein 2014	The evolutionary Stokes shift describes how, subsequent to fixation of an amino acid variant, epistatic interactions cause shifts in amino acid preferences, tending to make the newly resident amino acid more favorable.
Sikosek and Chan 2014	The simulation showed that an enforced destabilizing mutation (which could arise in real proteins owing to functional constraints such as the need to preserve an active site) can be compensated by subsequent mutations, thus increasing the future viability of the already-mutated residue at that same site and rendering the reverse mutation detrimental and therefore less probable.

Risso et al 2014	Indeed, recent computational analyses (Pollock et al. 2012) support the notion that preferences change after mutation in the direction of making the new amino acid more acceptable over time, an adjustment that is referred to as the “evolutionary Stokes shift.”
Goldstein et al 2015	Pollock et al. (2012) demonstrated how amino acid propensities at a site will adjust over time after a substitution, such that the resident amino acid (and others with similar physicochemical properties) tends to be the most favorable amino acid at that site, an effect they termed an “evolutionary Stokes shift.”
Shah et al 2015	In particular, we ask whether mutations that are nearly neutral when they fix subsequently become deleterious to revert later in the trajectory—a phenomenon that Pollock et al. (18) have called an “evolutionary Stokes shift.”
Echave et al 2016	evolutionary Stokes shift: when an amino acid substitution occurs at a site, its neighbours evolve more rapidly to accommodate the substitution.
Storz 2016	Similar findings reported in reference 87, this computational study demonstrates that individual amino acid substitutions at a given site can alter the amino acid propensities of other sites in the same protein; consequently, once a given substitution has occurred, the protein will tend to equilibrate to the newly altered structural context via substitutions at other sites.
Abriata et al 2016	As a more extreme consequence of coevolution effects, the “evolutionary Stokes shift”, i.e. the mechanism by which amino acid preferences at certain sites re-adapt to accepted changes at other sites [51], is also expected to blur correlations with amino acid descriptors.
McCandlish et al 2016	The first factor is coevolution between sites as suggested by, e.g., Pollock et al. (2012). As long as the derived allele is resident at the focal site, it forms part of the genetic background for other substitutions, and this causes the population to tend to spend more time at genotypes where the derived allele is selectively favored.
Goldstein and Pollock 2016	One aspect of this is the evolutionary ‘Stokes shift’, in which the rest of a protein adapts itself to a new amino acid resident at a given site, tending to make the resident amino acid more evolutionarily stable over time.

Echave and Wilke 2017	In addition, substitutions become entrenched over time: A mutation that was nearly neutral when fixed becomes increasingly deleterious to revert as subsequent substitutions accumulate. Entrenchment had previously been demonstrated in a stability-based simulation study by Pollock et al. (56).
Rodrigue and Lartillot 2016	Furthermore, under epistasis, fluctuations at a given site are such that the fitness landscape at that site tends to change so as to stabilize the fitness of the current state, a phenomenon referred to as entrenchment (Shah et al. 2015), or evolutionary Stokes shift (Pollock et al. 2012).
Bastolla et al 2017	Initially slightly destabilizing substitutions progressively become better adapted to their new background and more unlikely to revert (entrenchment), a phenomenon that is assimilated to the Stokes effect in atomic radiation.
Goldstein and Pollock 2017	These models exhibit a phenomenon called the evolutionary Stokes shift, the tendency for newly resident amino acids at a site to be stabilized, or ‘entrenched’ over evolutionary time following a substitution. We have also observed a tendency of the new amino acid to be pre-stabilized before the substitution by chance or contingency.
Teufel and Wilke 2017	A number of recent studies have focused on understanding the influence of epistasis at the level of position-specific substitutions along a protein sequence [6,13,19,35]. A core concept in these works is that substitutions that were neutral or nearly neutral at the time of fixation become entrenched over time, and the probability of reverting back to the substitution’s predecessor diminishes as additional mutations accumulate [19].
Flynn et al 2017	We observe strong epistatic effects. The primary mutations are destabilizing in the context of the wildtype background, but become stabilizing on average as other resistance mutations accumulate in the background, similar to the concept of entrenchment in systems biology (Pollock et al. 2012; Gong et al. 2013; Shah et al. 2015).
Haddox et al 2018	One idea that has recently gained support in the protein-evolution field is that substitutions become ‘entrenched’ by subsequent evolution (Pollock et al., 2012; Shah et al., 2015; Starr et al., 2017). Entrenchment is the tendency of a mutational reversion to become increasingly unfavorable as a sequence evolves.

Jiang et al 2018	Our findings are consistent with recent works on evolutionary entrenchment (Pollock et al. 2012; McCandlish et al. 2015, 2016; Shah et al. 2015; Goldstein and Pollock 2016), which argue that the propensity of a protein to acquire position-specific substitutions varies over time, as previously accumulated mutations become entrenched in the protein structure and slowly alter the constraints imposed on other amino acids in the structure.
Starr et al 2018	Conversely, a neutral mutation—which by definition is initially reversible to the ancestral state without fitness cost—may become entrenched by a subsequent restrictive substitution that renders the ancestral state deleterious (1, 4, 5); reversal of the entrenched mutation would then be unlikely unless the restrictive substitution were itself reversed or another permissive substitution occurred.
Teufel et al 2018	The fitness landscapes at these sites tend to change to stabilize the current state [62,63], a phenomenon referred to as entrenchment or an evolutionary Stokes shift.
Posfai et al 2018	In particular, previous simulation studies have described complex temporal dynamics at individual sites, so that the set of tolerable amino acid mutations at any given site changes over time [13,15,16,44,45], a process that has been called an "evolutionary Stokes shift", and which is also related to the phenomena of "contingency and entrenchment" and the expectation that reversion rates will decrease over time [15,45–47].
Castiglione and Chang 2018	This observation is consistent with an intramolecular evolutionary process known as entrenchment (Pollock et al., 2012; Goldstein and Pollock, 2017; Shah et al., 2015), where functionally favourable amino acid residues compensating for an original mutation tend to become fixed, thus mutually entrenching favourable amino acids at each position within the coevolving network.
Guin and Gruebele 2019	This is because a mutation, M, fixed at any given time interacts with many other mutations that occur at later times, and the deletion of M negatively affects all other mutations that are contingent on M. This phenomenon has been referred to as an "evolutionary Stokes shift"

Biswas et al 2019	Because the beneficial effects of the associated mutations depend on the primary mutation, with the accumulation of (accessory) mutations, the reversion of the primary mutation can become increasingly deleterious, leading to a type of evolutionary ‘entrenchment’ of the primary mutation (Pollock et al., 2012; Shah et al., 2015; McCandlish et al., 2016). The entrenchment effect on a primary mutation can be very strong on average, and is in fact, modulated by the collective effect of the entire sequence background.
Popova et al 2019	Indeed, substitutions at epistatically interacting sites are expected, on average, to increase the fitness of the incumbent allele—a phenomenon previously referred to as the Stokes shift (15), leading to entrenchment (6, 7, 14, 15) of the current allele.
Northover et al 2020	When physical changes within a molecule do occur, corresponding com- pensatory changes can occur which alleviate the deleterious effects of a mutation. These compensatory changes ensure that the newly substituted amino acid becomes the preferred amino acid for the residue in which it is located [61, 70].
de la Paz et al 2020	Pollock et al. (11) observed, using an energetic model, that when a change is made to a site, the compensatory changes to the rest of the sequence tend to make it favorable for that particular amino acid to remain in that position. They found that the $\Delta\Delta G$ for a mutation in a sequence has the opposite sign, but does not have the same magnitude when the inverse change is made to the sequence once it has evolved. This effect was named an evolutionary Stokes shift (11)
Tomaska and Nosek 2020	An important discovery stemming from the work on protein evolution is the "entrenchment" of resident amino acids and the "evolutionary Stokes shift" facilitating neutral amino acid replacements (Pollock et al. 2012; Shah et al. 2015). The "Stokes shift" concept is also broadly applicable to scenarios in which several distinct components of a system must co-evolve (e.g., niche spaces of species in an ecosystem) thus alluding to Waddington’s concept of genetic assimilation.

Table S2. Assessing robustness of results to simulation settings. Reported are the percentage of substitutions where the metric (M_{SLR} or M_{AMI}) values were negative. P-values as based on the Binomial test where the null hypothesis assumes an equal percentage of positive and negative values. Results based on simulations of the 1qhw protein.

Simulation	Number of trials	Number of windows	% M_{SLR} < 0	P-value	% M_{AMI} < 0	P-value
(1) Ne = 1e2	500	36,934	51.8	<0.001	42.4	<0.001
(2) Ne = 1e6	50	3,659	50.0	1.0	40.8	<0.001
(3) Thermodynamic propensity	500	36,934	51.2	<0.001	42.2	<0.001
(4) Longer equilibration phase	50	4,268	49.3	0.40	39.6	<0.001
(5) # subs = 5000	50	88,229	51.1	<0.001	34.3	<0.001
(6) Ne = 1e6 & # subs = 5000	50	85,643	51.2	<0.001	34.8	<0.001

Notes:
(1) Original simulation reported in the main manuscript.
(2) Same settings as (1) but with higher Ne
(3) Propensity was calculated as described in Pollock et al 2012.
(4) Included an additional 4500 substitutions in the equilibration phase.
(5) Ran the simulation for 5000 (rather than 500) substitutions.
(6) Longer simulations (5000 subs) with larger Ne (= 1e6)

Table S3. Average autocorrelation values in resident amino acid propensities or propensity changes following nonsynonymous substitutions. Reported are the mean values across sites and windows.

	Propensities	Propensity changes
1QHW	0.50	-0.22
1PEK	0.52	-0.24
2PPN	0.46	-0.21

Table S4. Differences in average rate of change between substitutions experiences evolutionary Stokes and anti-Stokes shifts. Reported are the P-values based on Welch's t-test. Substitutions are classified as undergoing an evolutionary Stokes (or anti-Stokes shift) if the corresponding metric value was greater than (or less than) zero.

	**1qhw**		**1pek**		**2ppn**	
	Difference in means	P-value	Difference in means	P-value	Difference in means	P-Value
**$\|M2_{SLR}\| - \|M1_{SLR}\| / T_{res}$**	-1.7e-6	0.55	1.3e-6	0.58	-1.8e-6	0.59
**$\|M2_{AMI}\| - \|M1_{AMI}\| / T_{res}$**	-1.3e-6	0.62	2.3e-6	0.31	-2.2e-6	0.41

Note: Difference calculated as mean value of anti-Stokes shifts minus mean for Stokes shifts.
T_{res} is the amino acid residency time measured as the number of substitutions during which an amino acid was resident at the site.
$M1_X$ represents the value of each metric calculated over the first half of the amino acid residency.
$M2_X$ is the value of the same metric calculated over the second half.

Table S5. Differences in the average metric value based on position in the protein (exposed versus buried sites). Reported P-values are based on Welch's t-test. Null hypothesis is that both exposed and buried sites have identical mean values.

	**1qhw**		**1pek**		**2ppn**	
	Difference in means	P-value	Difference in means	P-value	Difference in means	P-Value
**M_{SLR}**	-4.2e-5	0.16	-7.5e-5	0.005	7.5e-5	0.063
**M_{AMI}**	-0.006	<0.001	-0.010	<0.001	-0.008	<0.001

Note: Difference calculated as mean value at exposed sites minus mean at buried sites.
A site is considered buried if its relative solvent accessibility (RSA) ≤ 0.05, and is exposed if RSA > 0.05.

Table S6. Differences in the average rate of change based on position in the protein (exposed versus buried). Reported P-values are based on Welch's t-test.

	**1qhw**		**1pek**		**2ppn**	
	Difference in means	P-value	Difference in means	P-value	Difference in means	P-Value
**$\|M2_{SLR}\|$- $\|M1_{SLR}\|$ / T_{res}**	3.1e-6	0.39	1.0e-6	0.72	-4.9e-6	0.35
**$\|M2_{AMI}\|$- $\|M1_{AMI}\|$ / T_{res}**	-1.2e-7	0.97	3.8e-6	0.17	-2.3e-7	0.58

Note: Difference calculated as mean value at exposed sites minus mean at buried sites.
A site is considered buried if its relative solvent accessibility (RSA) ≤ 0.05, and is exposed if RSA > 0.05.

Table S7. Algorithm for obtaining sequences with high fitness (> 0.99) values**,** from Youssef et al 2020.

1. Start at random amino acid sequence
2. Calculate the site-specific fitness landscape at all sites
3. If a single step uphill move is possible (i.e. beneficial mutation), then randomly choose the next substitution from the set of single amino acid changes that will increase fitness
4. If no uphill move is possible (i.e. local maximum), then randomly choose 20 sites and substitute them to the fittest amino acid at that site
5. Repeat 2-4 until fitness is greater than 0.99

Table S8. Set of alternative structures used to estimate the free energies in the unfolded state.

1cnz	1hz4	1moq	1o88	1svm
1dmh	1i4w	1mty	1oc7	1t5j
1E_19	1iom	1n00	1odm	1t5o
1ek6	1ir6	1nbf	1ojj	1to6
1esd	1jfb	1nd6	1pby	1uby

1ga6	1jil	1nsz	1pfk	1umd
1gwu	1jix	1o4s	1qo0	1v6s
1gyh	1jj2	1o7j	1qop	1wch
1kwf	1jkm	1m4l	1rkd	1wer
1l5o	1jl5	1mkf	1sbp	1wkr
2bbv	1jub	2mas	3sil	1woh

Supplementary Figures:

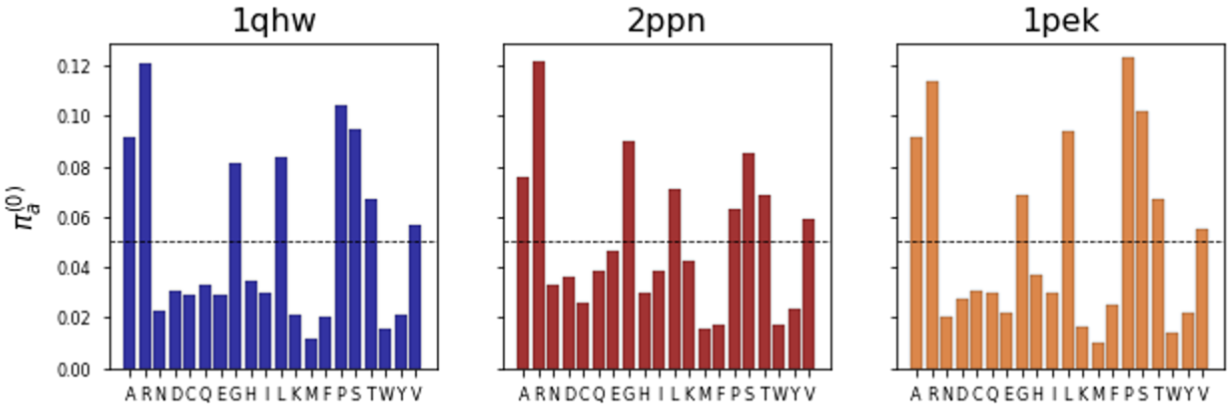

Figure S1. The expected amino acid frequencies in the absence of selection but accounting for underlying mutational biases. The dotted line represents the expected frequency values in the absence of mutational biases and assuming all amino acids have the same number of codon aliases (=1/20).

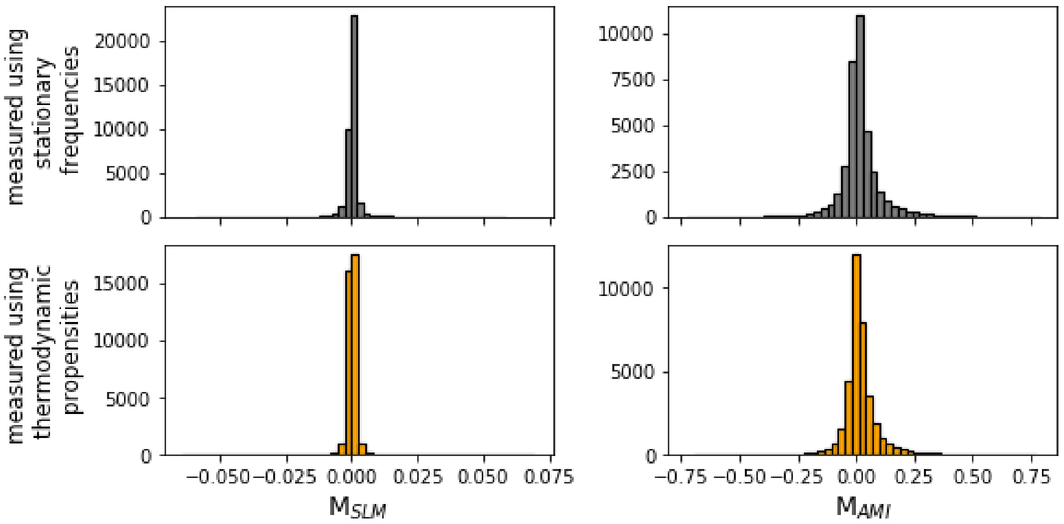

Figure S2. Metric distributions are comparable under both definitions of propensity. The distribution of metrics M_{SLR} and M_{AMI} where propensity was calculated as the stationary frequency of the amino acid (using equation 1 in the main text; top row), or where propensity is a thermodynamic based quantity as described in Pollock et al 2012 (bottom row). The thermodynamic based propensity differs from the stationary frequency by not accounting for underlying mutational biases. Comparison between the mean metric value between propensity and frequency analysis using Welch's t-test was not statistically significant (all P-values > 0.05). Plotted are the results based on 500 simulations of the 1qhw protein.

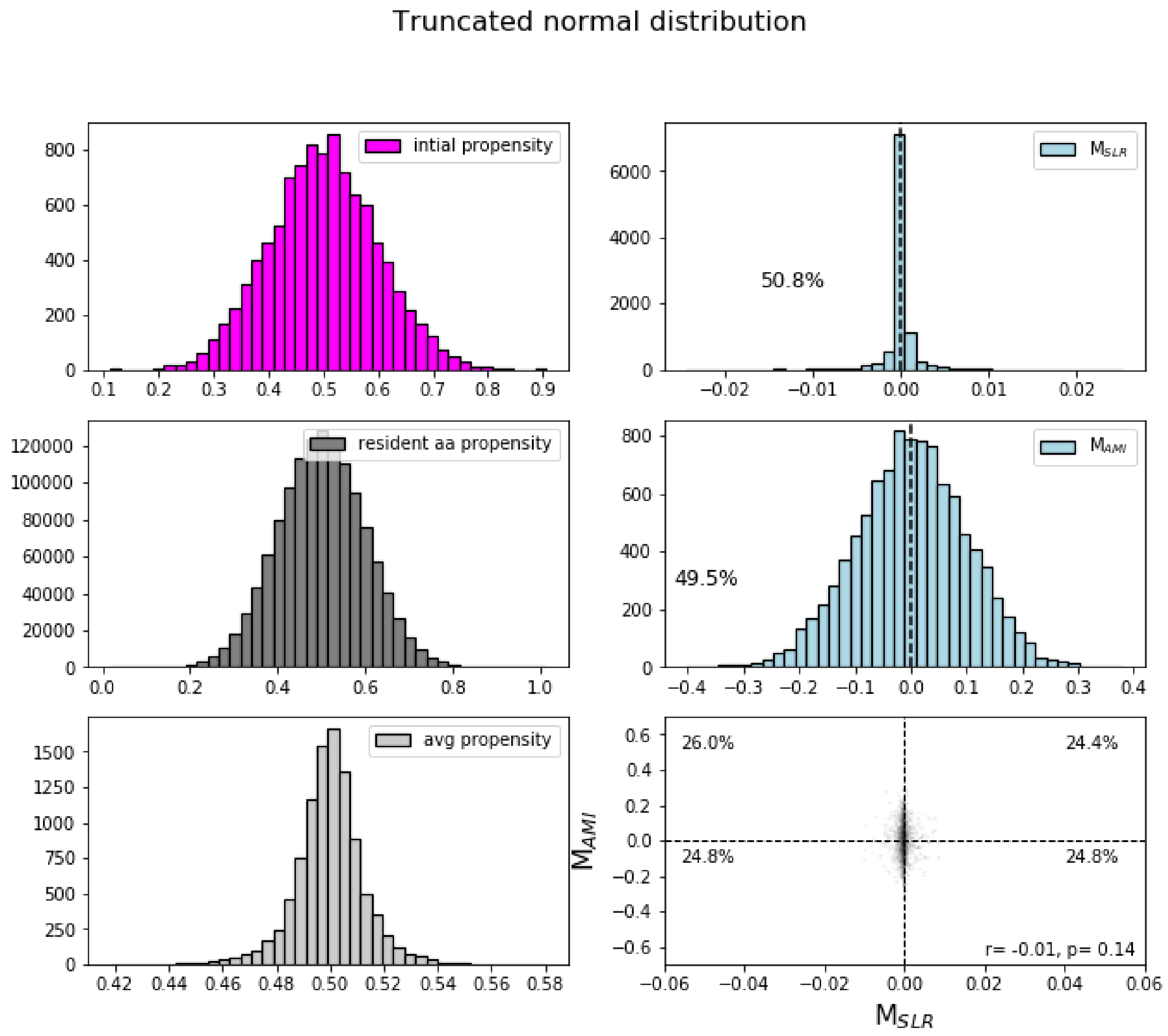

Figure S3. Equal number of negative and positive shifts in propensities when propensities are sampled from truncated normal distribution.

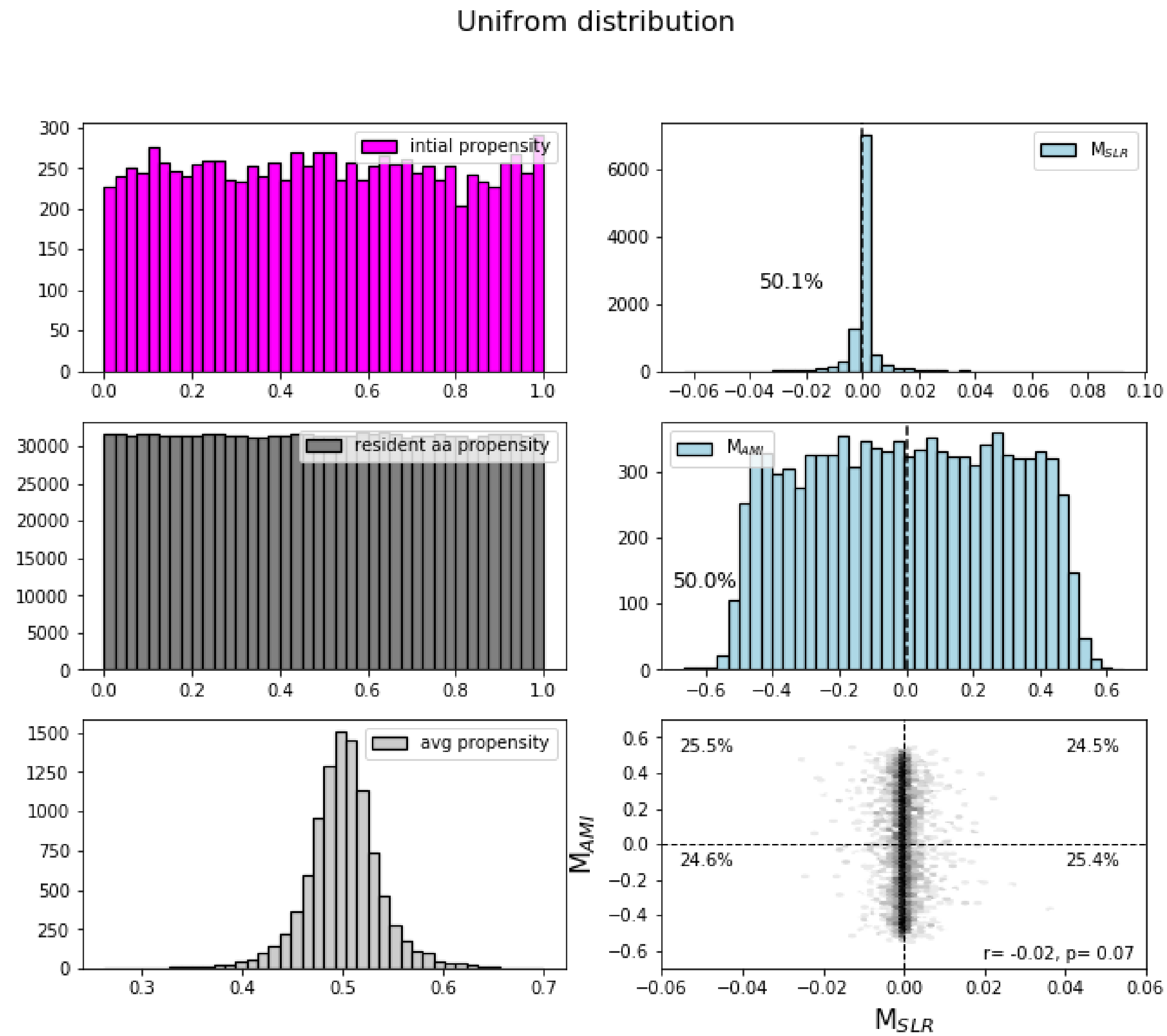

Figure S4. Equal number of negative and positive shifts in propensities when propensities are sampled from uniform distribution.

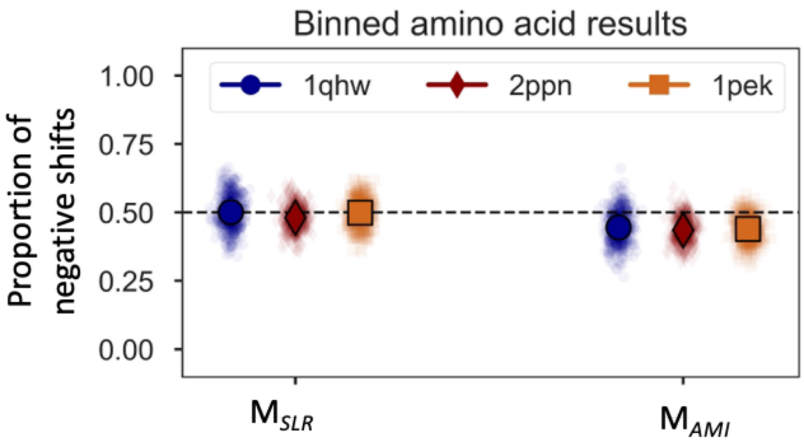

Figure S5. Proportion of evolutionary anti-Stokes shifts considering shifts in propensities in groups of amino acids. Amino acids were grouped as: AST, C, DE, FY, GN, HQ, IV, KR, LM, P, W (Susko and Roger 2007). Bins represent amino acids which tend to interchange rapidly and have similar chemical properties. Evolutionary shifts were calculated based on the sum of propensities for all amino acids in a specific bin. The summed group propensites decreases in approximately half of substitutions based on metrics M_{SLR} and M_{AMI}.

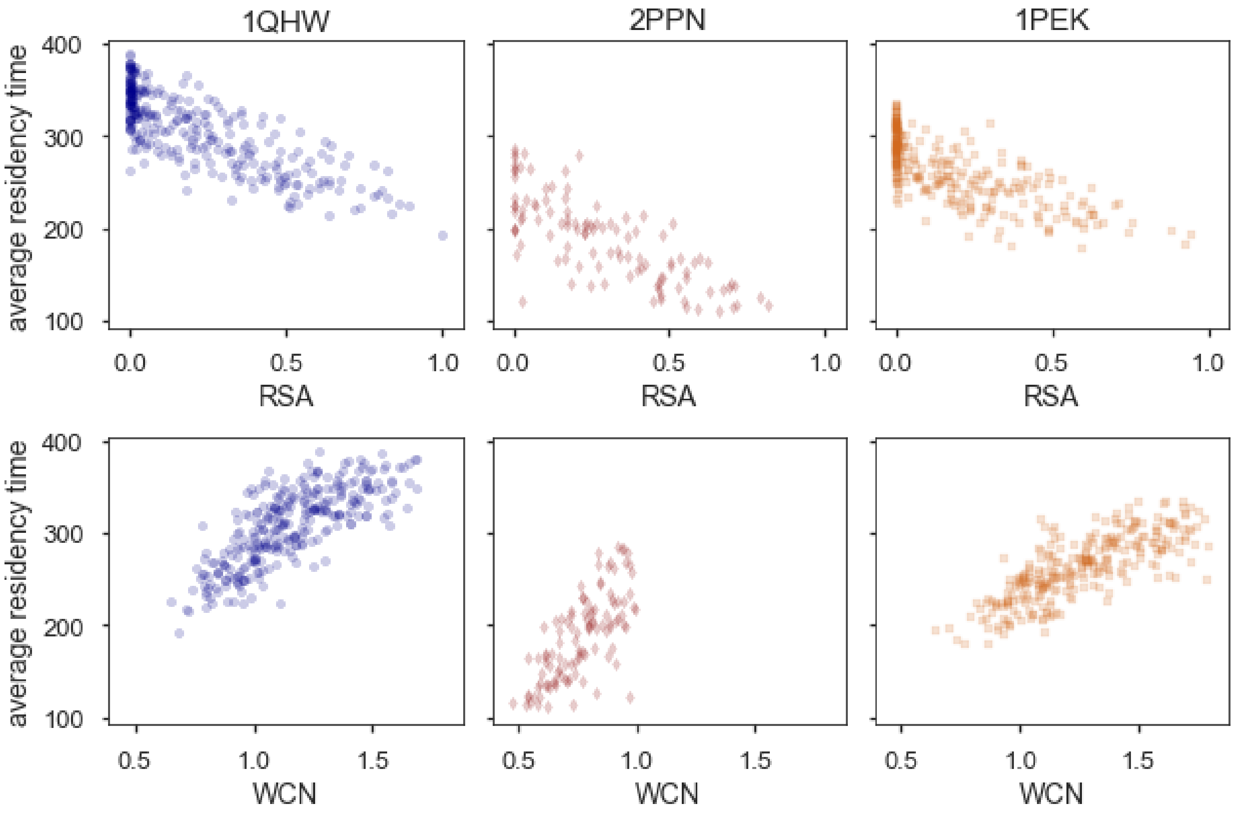

Figure S6. Relationship between average amino acid residency time and location in the protein. Plotted are the correlations with relative solvent accessibility (RSA, top row), and weighted contact number (WCN, bottom row) for three proteins (1qhw, 2ppn, 1pek).

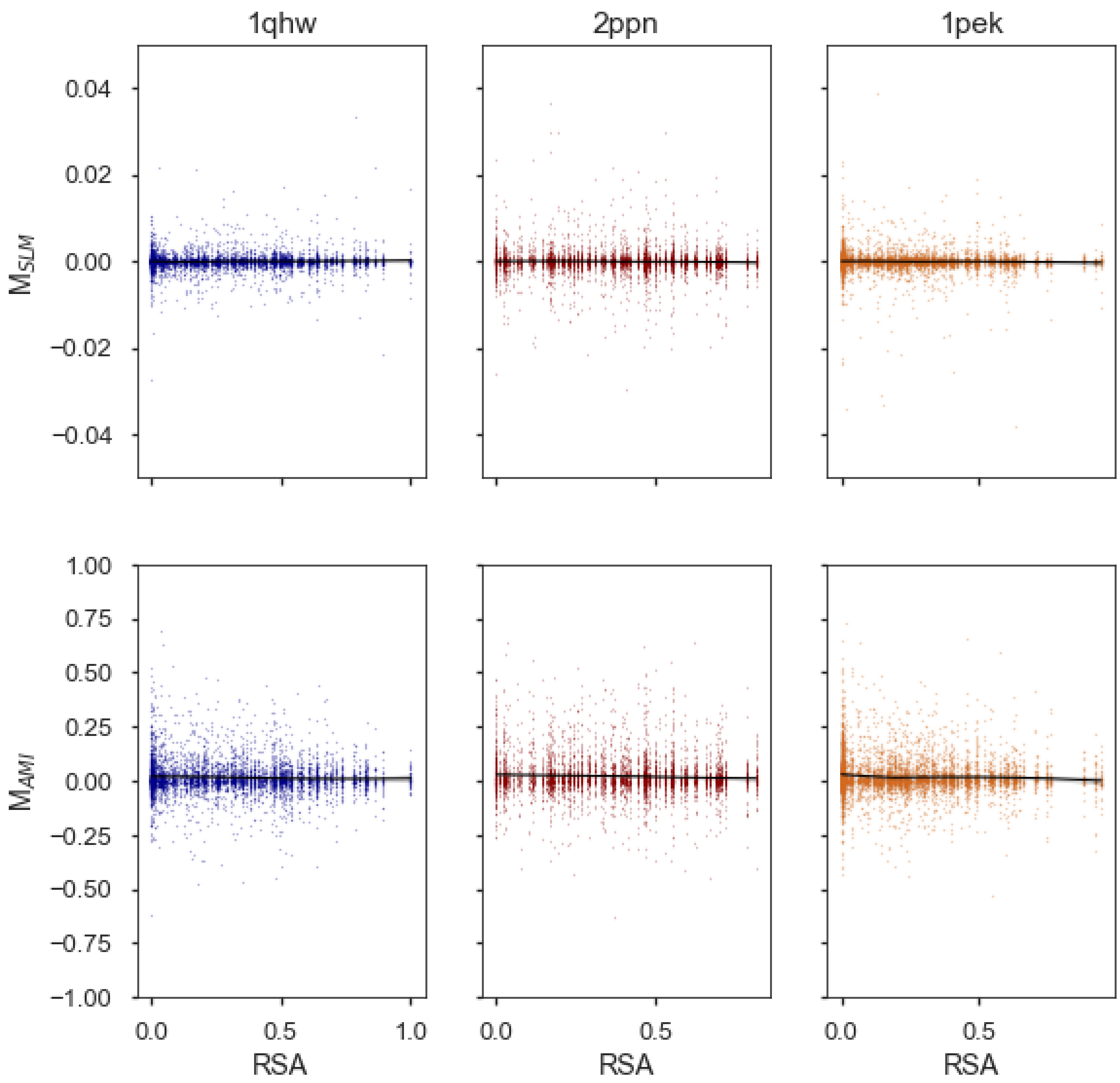

Figure S7. Relationship between metrics of evolutionary shifts (M_{SLR} and M_{AMI}) and relative solvent accessibility (RSA). Plotted are 5,000 randomly sampled points. Black line represents loess regression. Grey area is the prediction interval.

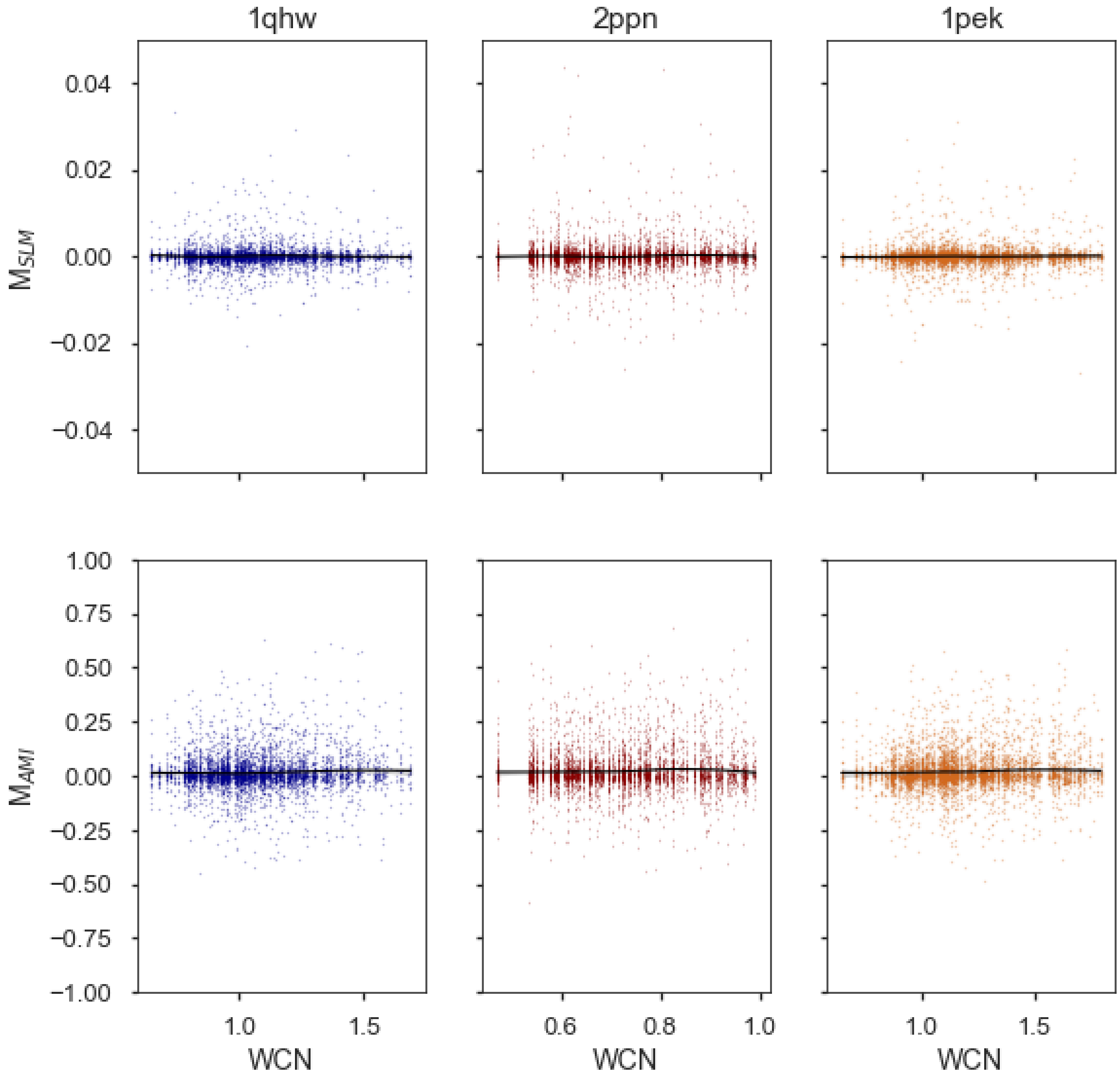

Figure S8. Relationship between metrics of evolutionary shifts (M_{SLR} and M_{AMI}) and weighted contact number (WCN). Plotted are 5,000 randomly sampled points. Black line represents loess regression. Grey area is the prediction interval.

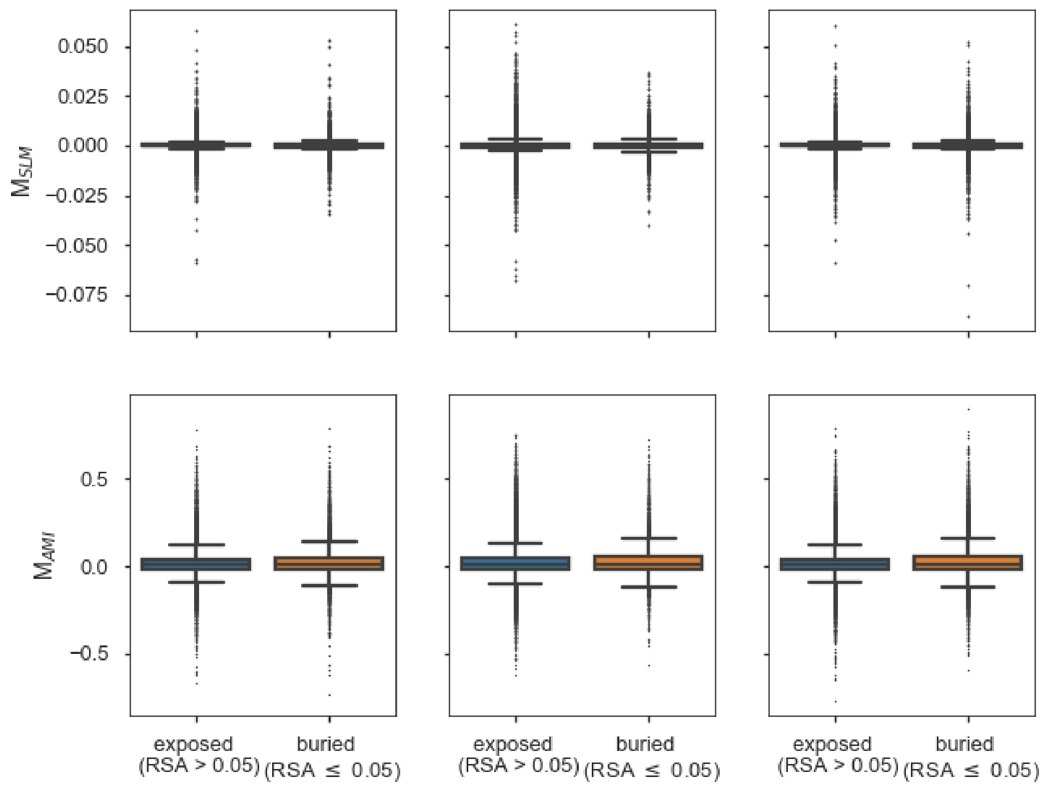

Figure S9. Evolutionary shifts in propensities occur with similar frequency and magnitude at exposed and buried sites. Sites are considered exposed if their relative solvent accessibility (RSA) is > 0.05, and are considered buried if RSA <= 0.05.

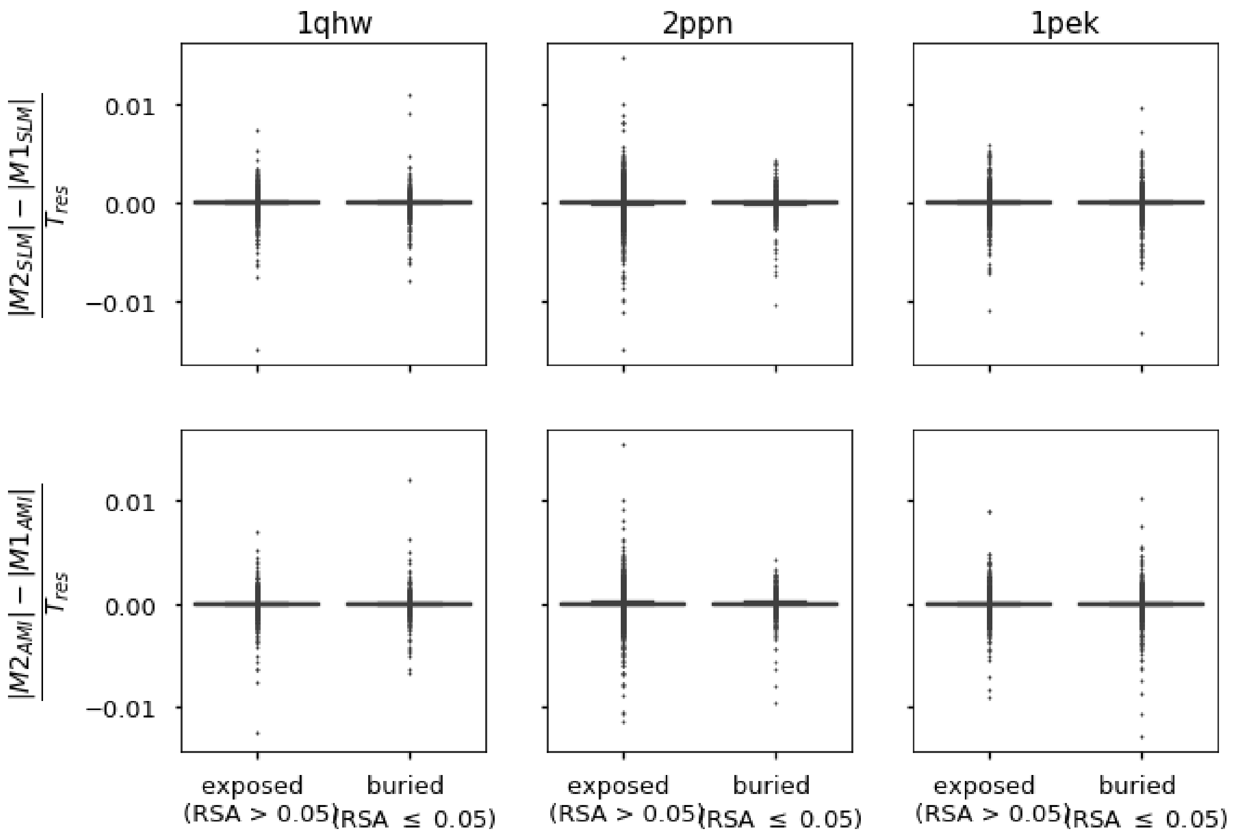

Figure S10. The rates of acceleration in evolutionary shifts are similar for buried and exposed sites. Sites are considered exposed if their relative solvent accessibility (RSA) is > 0.05, and they are considered buried if RSA <= 0.05.

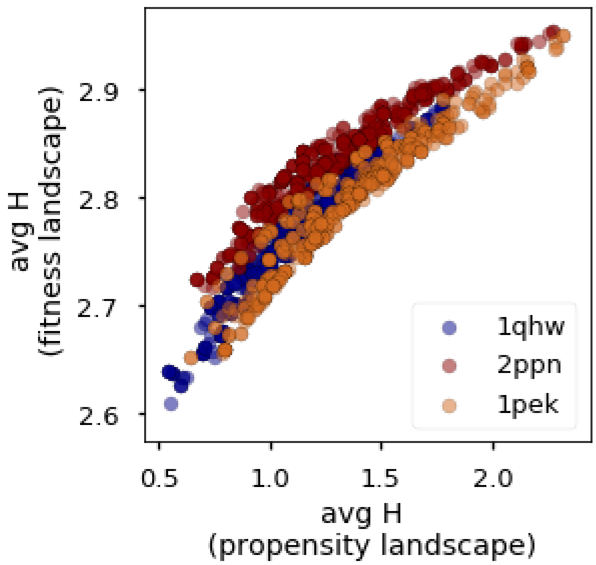

Figure S11. Relationship between the Shannon entropy of a propensity landscape compared with the entropy of the fitness landscape. Entropy of a landscape is calculated using equation (7) in the main text. To calculate the entropy of a fitness landscape, $\pi_a^h(s)$(the propensity for amino acid a at site h given background sequence s) is replaced with $f_a^h(s)$(the fitness of amino acid a at site h given background sequence s). Reported are the average entropy values over all sites given a particular background sequence from a single simulation trial for each of three protein structures (1qhw, 2ppn, and 1pek).

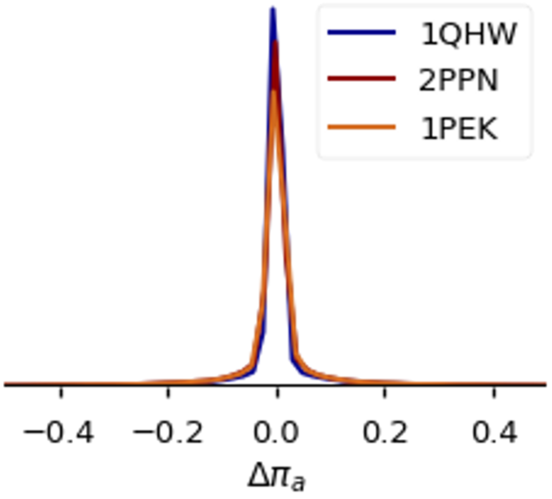

Figure S12. Distributions of the observed changes in resident amino acid propensities ($\Delta\pi_a^h$) following each substitution. Plotted are the results from 500 protein-specific simulations of three protein structures: 1qhw, 1pek, and 2ppn.
