# Supplementary material for: Evolution of Amino Acid Propensities under Stability-Mediated Epistasis
Source: Mol Biol Evol. 2022 Feb 4;39(3):msac030. doi: 10.1093/molbev/msac030 (PMC8896634; doi:10.1093/molbev/msac030)
Supplement: msac030_Supplementary_Data [file msac030_supplementary_data.zip › Supp_text.pdf]

Supplementary text

0.1 Autocorrelated model

Algorithm

Here we describe the algorithm for generating propensities with a fixed autocorrelation and keeping the marginal distribution of propensities equal to the empirical distribution. Let F be the empirical distribution of propensities observed throughout the stability informed simulations, and let ρ be the desired autocorrelation between propensity values estimated from the stability simulations. Let $\{y_0, y_1, \dots y_n\}$ denote the generated propensities over a given window of size n.

1. Generate initial propensity y_0 from the empirical distribution F.
2. For $j = 1 \dots n$,
    - 2.1 Generate $x_i = 0$ or 1 such that $P[X_j = 1] = p$ (where $p = \rho$ as argued below)
    - 2.2 If x_j =1 then
        - 2.2.1 set $y_j = y_{j-1}$
    - 2.3 else
        - 2.3.1 generate y_j from F

Proof by induction:

Here we demonstrate that the marginal distribution of propensities from the above algorithm will be equal to the empirical distribution F. Since y_0 is generated from F, $P[Y_j \leq y] = F(y)$ is true for $j = 0$. Suppose that it is true for $j = i$, that is $P[Y_i \leq y] = F(y)$. Then

$$\begin{aligned}
P[Y_{i+1} \leq y] &= P[Y_{i+1} \leq y | X_{i+1} = 1] * p + P[Y_{i+1} \leq y | X_{i+1} = 0] * (1 - p) \\
&= P[Y_i \leq y] * p + P[Y_{i+1} \leq y | X_{i+1} = 0] * (1 - p) \\
&= F(y) * p + P[Y_{i+1} \leq y | X_{i+1} = 0] * (1 - p) \\
&= F(y) * p + F(y) * (1 - p) \\
&= F(y)
\end{aligned}$$

Choice of p

Let μ and σ^2 denote the mean variance of the propensities determined from F. The covariance of Y_j and Y_{j-1} is

$$\text{Cov}(Y_j, Y_{j-1}) = \text{E}[Y_j Y_{j-1}] - \mu^2$$

where

$$\begin{aligned} \text{E}[Y_j Y_{j-1}] &= p\text{E}[Y_{j-1}^2] + (1-p)\mu^2 \\ &= p(\sigma^2 + \mu^2) + (1-p)\mu^2 \\ &= p\sigma^2 + \mu^2 \end{aligned}$$

then setting ρ to the correlation gives

$$\begin{aligned} \rho &= \text{Cov}(Y_j, Y_{j-1})/\sigma^2 \\ &= [p\sigma^2 + \mu^2 - \mu^2]/\sigma^2 \\ &= p \end{aligned}$$

0.2 Is the process at equilibrium?

Sequence space is vast and randomly sampling sequences rarely produces viable proteins. Starting the simulation in such dire conditions leads to evolution randomly drifting between low fit sequences. These are not the dynamics we aim to assess. Instead, we aim to assess the dynamics at mutation-selection-drift equilibrium. Equilibrium is achieved when protein fitness, or more precisely its stability (ΔG), remains relatively constant. The results reported in the main text are based on 500 protein-specific simulations, each evolved for 500 substitutions. To assess if the process is at equilibrium, we performed additional simulations with the number of substitutions equal to 5,000 and 15,000. Due to intensive computational demand, we were only able to perform 50 and 12 replicates respectively using the 1qwh protein structure. We expect these analyses to generalize to the other structures since, as can be seen from the main text, all of the reported results are agnostic to the protein structure used. The distribution of stability values were similar despite increasing the number substitutions (average stability value of approx -3.7 kcal/mol with Ne = 1e2, and approx -9 kcal/mol with Ne = 1e6), indicating

that the process is at equilibrium (Figure ST1). Furthermore, allowing for a longer equilibration phase prior to conducting the simulations did not alter our results (Table S1, supplementary tables).

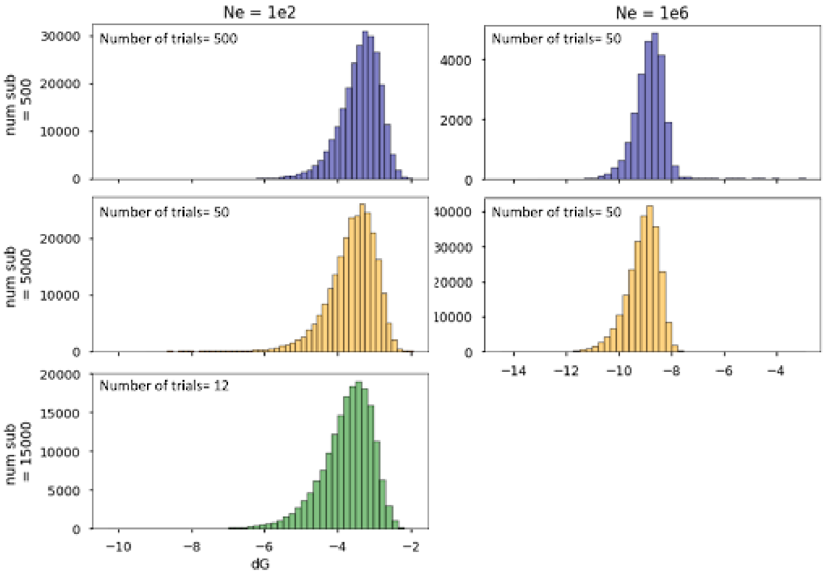

Figure ST1: Distribution of stability values given different effective population sizes (columns) and different simulation lengths (rows).

0.3 Does Ne affect the results?

The effective population size (Ne) plays an important role in tuning the relative influences of selection and drift on the evolutionary process. However, Goldstein and Pollock (2017) have argued that substitution rates and the evolutionary Stokes shift do not depend on Ne. We found that while Ne dictates the average protein stability observed at equilibrium (so that the average stability value is approx -9 kcal/mol, compared to approx -3.7 kcal/mol with Ne = 100; figure ST1), metric distributions, for both MAMI and MSLR, were largely indistinguishable between simulations where Ne was 1e2 or 1e6 (figure ST2).

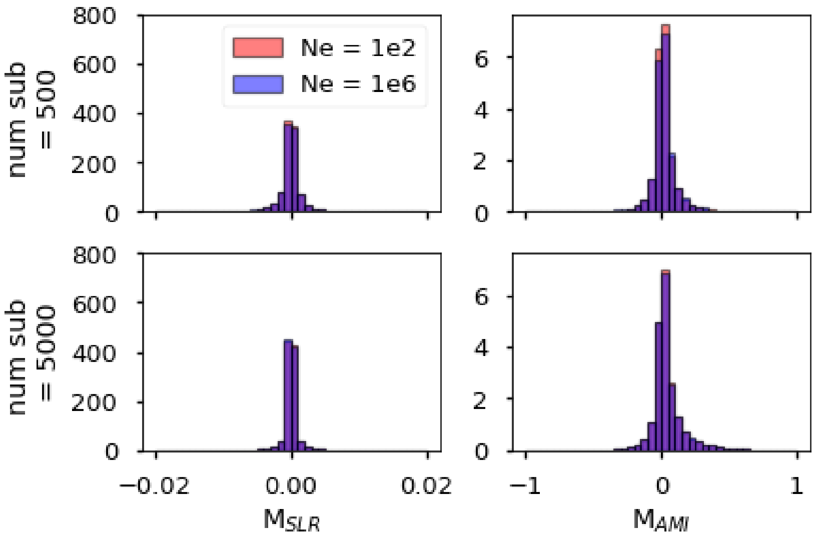

Figure ST2: Distribution of metrics (M_{SLR} and M_{AMI}) given different effective population sizes (Ne = 1e2 and 1e6) were indistinguishable.
